# A cytoplasmic RNA virus generates functional viral small RNAs and regulates viral IRES activity in mammalian cells

**DOI:** 10.1093/nar/gku952

**Published:** 2014-10-28

**Authors:** Kuo-Feng Weng, Chuan-Tien Hung, Po-Ting Hsieh, Mei-Ling Li, Guang-Wu Chen, Yu-An Kung, Peng-Nien Huang, Rei-Lin Kuo, Li-Lien Chen, Jing-Yi Lin, Robert Yung-Liang Wang, Shu-Jen Chen, Petrus Tang, Jim-Tong Horng, Hsing-I Huang, Jen-Ren Wang, David M. Ojcius, Gary Brewer, Shin-Ru Shih

**Affiliations:** 1 Research Center for Emerging Viral Infections, College of Medicine, Chang Gung University, Taoyuan, Taiwan; 2 Center for Molecular and Clinical Immunology, College of Medicine, Chang Gung University, Taoyuan, Taiwan; 3 Graduate Institute of Biomedical Sciences, College of Medicine, Chang Gung University, Taoyuan, Taiwan; 4 Department of Biochemistry and Molecular Biology, Robert Wood Johnson Medical School, Rutgers University, Piscataway, NJ, USA; 5 Department of Computer Science and Information Engineering, College of Engineering, Chang Gung University, Taoyuan, Taiwan; 6 Department of Medical Biotechnology and Laboratory Science, College of Medicine, Chang Gung University, Taoyuan, Taiwan; 7 School of Medical Laboratory Science and Biotechnology, College of Medical Science and Technology, Taipei Medical University, Taiwan; 8 Department of Biomedical Sciences, College of Medicine, Chang Gung University, Taoyuan, Taiwan; 9 Molecular Medicine Research Center, Chang Gung University, Taoyuan, Taiwan; 10 Center of Infectious Disease and Signaling Research and Department of Medical Laboratory Science and Biotechnology, National Cheng Kung University, Tainan, Taiwan; 11 Health Sciences Research Institute and School of Natural Sciences, University of California, Merced, CA, USA; 12 Clinical Virology Laboratory, Chang Gung Memorial Hospital, Tao-yuan, Taiwan

## Abstract

The roles of virus-derived small RNAs (vsRNAs) have been studied in plants and insects. However, the generation and function of small RNAs from cytoplasmic RNA viruses in mammalian cells remain unexplored. This study describes four vsRNAs that were detected in enterovirus 71-infected cells using next-generation sequencing and northern blots. Viral infection produced substantial levels (>10^5^ copy numbers per cell) of vsRNA1, one of the four vsRNAs. We also demonstrated that Dicer is involved in vsRNA1 generation in infected cells. vsRNA1 overexpression inhibited viral translation and internal ribosomal entry site (IRES) activity in infected cells. Conversely, blocking vsRNA1 enhanced viral yield and viral protein synthesis. We also present evidence that vsRNA1 targets stem-loop II of the viral 5′ untranslated region and inhibits the activity of the IRES through this sequence-specific targeting. Our study demonstrates the ability of a cytoplasmic RNA virus to generate functional vsRNA in mammalian cells. In addition, we also demonstrate a potential novel mechanism for a positive-stranded RNA virus to regulate viral translation: generating a vsRNA that targets the IRES.

## INTRODUCTION

Cells produce small RNAs, which are noncoding RNAs 20–30 nucleotides (nt) in length (1). These small RNAs can fine-tune the biological functions of cells by modulating gene expression and modifying the genome (2,3). For example, endogenous microRNAs (miRNAs) regulate specific gene expression and control the associated downstream activities (2). Another type of cellular small RNAs, PIWI-interacting RNAs (piRNAs), maintain genomic integrity by preventing the invasion of transposable elements (3).

Mammalian cells produce numerous small RNAs via a canonical miRNA biogenesis pathway that involves nuclear processing by an RNase III-type protein, Drosha, and subsequent cytoplasmic processing by another RNase III-type protein, Dicer (1). Viruses that replicate in the nucleus, such as deoxyribonucleic acid (DNA) viruses and retroviruses, can produce their own small RNAs through the canonical miRNA biogenesis pathway. These virus-derived small RNAs (vsRNAs) either fine-tune viral replication or inhibit antiviral mechanisms in infected cells (4–6). Alternatively, Dicer enzymes in plant and insect cells process the genome of a cytoplasmic RNA virus into small RNAs. Infected cells use these vsRNAs as an antiviral defence mechanism to reduce viral replication through RNA interference (7). Conversely, West Nile virus uses Dicer in mosquito cells to produce miRNA-like vsRNAs for the benefit of the virus (8). However, similar mechanisms for generating vsRNA and RNA-based defences against cytoplasmic RNA viruses in mammals require further research (2,9–10).

Recent studies have shown that cytoplasmic RNA viruses can induce non-canonical cytoplasmic miRNA biogenesis pathways in mammalian cells (11,12). For example, an engineered Sindbis virus with a primary miRNA hairpin in its subgenomic RNA generated functional miRNA through a Dicer-dependent, DGCR8-independent pathway (12,13). These studies have suggested that a cytoplasmic RNA virus containing a primary miRNA-like hairpin may be capable of producing vsRNA through its own structured RNA in infected mammalian cells. In addition, deep sequencing techniques have been used to identify vsRNAs and siRNAs in mammalian cells infected with cytoplasmic RNA viruses (14–16). However, the functions of these vsRNAs are still debated (17).

Similar to poliovirus, enterovirus 71 (EV71) is a positive-stranded RNA virus that replicates in the cytoplasm. EV71 outbreaks have occurred worldwide, and EV71 infection is associated with severe neurological diseases and high mortality rates (18,19). The 5′ untranslated region (5′UTR) of the EV71 genomic RNA is highly structured (20,21); it contains a cloverleaf structure that is essential for viral RNA replication and an internal ribosomal entry site (IRES) that is responsible for viral translation (22,23). Because positive-stranded viruses use the same RNA template for both translation and replication, viruses must regulate their translation (or IRES activity) (24,25). Recently discovered proteins called IRES trans-acting factors (ITAFs) can regulate EV71 IRES activity (19,26–28).

In this study, we showed that a cytoplasmic positive-stranded RNA virus generated functional vsRNAs in mammalian cells. One vsRNA (vsRNA1) down-regulated viral translation by targeting the stem-loop II region of the viral IRES. This study demonstrated a novel mechanism by which virus self-regulates its translation by generating a RNA-based ITAF.

## MATERIALS AND METHODS

### Deep sequencing and data analysis

SF268 (human glioblastoma) cells were mock-infected or virus-infected with Enterovirus 71 strain Tainan/4643/98 (GenBank accession number: AF304458.1) at a moi of 40. After 6 h post-infection (p.i.), the total RNA was extracted with a TRIzol reagent (Invitrogen) according to manufacturer instructions. The integrity and quality of the total RNA was evaluated using an Agilent 2100 BioAnalyzer (Agilent Technologies). Forty micrograms of RNA were sent to the Beijing Genomics Institute (BGI) for Solexa analysis. Small RNAs under 50 bases were PAGE-purified and ligated with a pair of Solexa adaptors to their 5′ and 3′ ends. The small RNAs were then reverse transcribed and amplified by PCR using a pair of adaptor primers. The resulting cDNA library then underwent cluster generation and sequencing analysis using the Illumina HiSeq 2000 sequencing system (Illumina). Raw sequencing data were analyzed using a CLC Genomics Workbench 4.7 (CLC Bio). Raw reads were filtered by discarding low-quality reads and removing adaptor sequences to generate clean and usable reads with sizes ≥15 nt and ≤50 nt. To discard unique sequences originating from cellular miRNA and other non-coding RNA, including rRNA, tRNA, small nuclear RNA (snRNA) and small nucleolar RNA (snoRNA), clean reads were first mapped to the miRBase version 18 (www.mirbase.org) and Homo_sapiens.GRCh37.65.ncrna (www.ensembl.org). The remaining reads were then mapped to the EV71 genome (strain 4643), and only the reads that matched the EV71 genome perfectly were selected for further analysis. The mapping results were exported from CLC Genomics Workbench and were illustrated by Prism 5 (GraphPad).

### vsRNA detection in EV71-infected cells

SF268 and RD cells were mock-infected or infected with EV71/4642/MP4 at an MOI of 40. At 6 h p.i., small RNA fractions with sizes ≤ 200 nt were isolated and enriched using the mirVana miRNA Isolation Kit (Ambion). Ten microgram samples of small RNA were separated on 15% polyacrylamide/8M urea denaturing gel, electroblotted to GeneScreen Plus nylon membranes (PerkinElmer), and UV cross-linked for fixation. The oligonucleotides used to probe each vsRNA were 5′-TGATCGTTGATTTACAGCTTCTAAGTTAC-3′ for vsRNA1, 5′-TGATACTCAGTCCGGGGAAAC-3′ for vsRNA2, 5′-GTCGGTTCCGCTGCAGAGTTGCCCG-3′ for vsRNA3, 5′-TGAGAGTGATCACAGACTTCAG-3′ for vsRNA4 and 5′-AAACAGAAGTGCTTGATCA-3′ for 163–181 fragment. The oligonucleotide probes were generated by 5′-end-labelled with [α-^32^P]ATP using T4 polynucleotide kinase (Promega). Prehybridization and hybridization of the membrane were conducted with an ULTRAhyb Hybridization Buffer (Ambion) containing denatured salmon sperm DNA at 42°C. After probe hybridization, the probed membranes were washed twice with 2× SSC, and 0.1% SDS at 42°C for 15 min, and were then processed by autoradiography.

### Co-immunoprecipitation of Ago2 and miRNA-21 in infected cells

RNAs in cell lysates from EV71-infected SF268 cells were immunoprecipitated with anti-Ago2 antibody (ab32381, Abcam) at 4°C for 4 h. The co-precipitated RNAs were purified from the lysates after treated with DNase and Protease K. vsRNA1 and miR-21 in the co-precipitation were detected by northern-blotting using 5′^32^P-radiolabelled probes (5′-TGATCGTTGATTTACAGCTTCTAAGTTAC-3′ for vsRNA1 and 5′-TCAACATCAGTCTGATAAGCTA-3′ for miR-21).

### Plasmids and Constructs

Plasmids pT7-EV71-IRES and pGL3-EV71-5 -UTR-Fluc were constructed previously (28,29). The pGL3-EV71-5′ UTR-Fluc was adopted as the template for constructing mutant EV71-5′ UTR plasmids by using site-direct mutagenesis kit (Stratagene) and primers 5′-(TGTGGCACACCAGTCATACCTACTTCAAGCACTTCTGTTTCCCCG)-3′ and 5′-(CGGGGAAACAGAAGTGCTTGAAGTAGGTATGACTGGTGTGCCACA)-3 for mut 163–165, 5′-(AGTCATACCTTGATCAAGCACAAGTGTTTCCCCGGACTGAGTATC)-3′ and 5′-(GATACTCAGTCCGGGGAAACACTTGTGCTTGATCAAGGTATGACT)-3′ for mut 174–176, 5′-(TGTGGCACACCAGTCATACCTACTTCAAGCACAAGTGTTTCCCCGGACTGAGTATC)-3′ and 5′-(GATACTCAGTCCGGGGAAACACTTGTGCTTGAAGTAGGTATGACTGGTGTGCCACA)-3′ for mut 163–165+174–176, 5′-(GGCGACCATGGCAGTGGCTGCCAAGGCGGCCTGCCCATGGAGAAA)-3′ and 5′-(TTTCTCCATGGGCAGGCCGCCTTGGCAGCCACTGCCATGGTCGCC)-3′ for mut 367–369, 5′-(ATGGTGACAATCAAAAAGTTGAATCCATATAGCTATTGGATTGGCC)-3′ and 5′-(GGCCAATCCAATAGCTATATGGATTCAACTTTTTGATTGTCACCAT)-3′

for mut 617–619. The plasmid pGL3-Polio-5′-UTR-Flu was constructed as follows: the 5′ UTR of poliovirus was amplified by PCR using the poliovirus replicon (30) primer 5′-(CCGCTCGAGTAATACGACTCACTATAGGGAGATTAAAACAGCTCTGGGGTTG)-3′, which contains the T7 promoter sequence and primer 5′-(AATCAGACAATTGTATCATAATGGAAGACGCCAAAAACAT)-3′. The polio-5′ UTR combined with the firefly luciferase gene was subsequently amplified by primers 5′-(CCGCTCGAGTAATACGACTCACTATAGGGAGATTAAAACAGCTCTGGGGTTG)-3′ and 5′-(ATGTTTTTGGCGTCTTCCATTATGATACAATTGTCTGATT)-3′. The segment containing polio-5′ UTR and the firefly luciferase gene was then cloned into vector pGL3 by *Xho*I and *Mlu*I. Plasmid FLAG-Dicer, FLAG-Dicer-MUT and FLAG-Dicer-dsRBD were constructed as follows: the WT and mutant (MUT) human Dicer cDNA were generated using PCR from the plasmids containing Dicer cDNA (WT or with quadruple mutants on 44th and 110th amino acids in Dicer RNaseIII Domain a and Domain b) in a pDEST8 vector [pDEST8-Dicer-6His and pDEST8-Dicer 44AB/110AB) were gifts from Dr. Witold Filipowicz (31)] using 5′-(ACGACAAGCTTATGAAAAGCCCTGCTTTG)-3′ primer containing the *Hin*dIII site and 5′-(AGTCCAAGGGTTATCGATTCCATGGCTATA)-3′ primer containing a stop codon and a *Kpn*I site. Truncated Dicer cDNA with dsRBD domain deletion (ΔdsRBD), which lacks the 57 C-terminal amino acids, was generated from plasmid Dicer-pDEST8 by PCR using 5′-(CCTTGGTCTTTGACGGATTCCATGGCTATA)-3′ as the downstream primer. The generated cDNAs were digested by *Hin*dIII and *Kpn*I and inserted into the vector p3XFLAG-Myc-CMV-25 (Sigma-Aldrich) to form the FLAG-Dicer, FLAG-Dicer-MUT and FLAF-Dicer-ΔdsRBD plasmids. The plasmid for the EV71 replicon was generated as follows: the DNA fragment containing the T7 promoter, EV71-5′ UTR, and firefly luciferase (FLuc) gene was sub-cloned from pGL3-EV71-5′-UTR-Fluc into vector yT&A (Yeastern Biotech). The DNA fragment containing the EV71 P2-P3 polyprotein region was amplified from the EV71 infectious clone (32) and cloned into downstream of the FLuc gene in yT&A-EV71-5′ UTR-FLuc. To generate the vsRNA1 deletion mutant on the replicon plasmid, a set of primers, 5′-(TTCGGGGGAAGGGGAGTAAA)-3′ and 5′-(TAGCAGGTGTGGCACACCAG)-3′ were used for amplifying the DNA fragment from the EV71 replicon plasmid. The amplified DNA was treated with DpnI (New England BioLabs), and then treated with T4 DNA ligase (New England BioLabs). The EV71 Replicon plasmid containing the vsRNA1 deletion mutation was selected and confirmed through sequencing. Plasmid for shDicer was generated by annealing synthetic shDicer oligonucleotides (33) into the pSilencer 2.1-U6 hygro vector. The plasmid for shAgo2 was a gift from Dr. Shobha Vasudevan (34). To construct sponge-vsRNA1 plasmids, a DNA fragment contains 10 repeats of the sequence complement of vsRNA1 (5′-TGATCGTTGATTTACAGCTTCTAAGTTAC-3′) was cloned into pcDNA3.1(+) vector (Invitrogen) by *Han*dIII and *Eco*RI. To construct sponge-controlled plasmids, a DNA fragment contain 10 repeats of sequence scrambled (5′-TCCGTGCCAAGTTACTAGAAAAGTTCAAT-3′) was cloned into pcDNA3.1(+) in the *Bam*HI and *Xho*I restriction sites. The plasmid pT7-EV71-3′UTR was generated as fellows: The cDNA of EV71 3′UTR was amplified by PCR using a set of primers, 5′-(AACTTAAGCTTTAAATTTACAGTTTGTAACT)-3′ and 5′-(TGCAGAATTCGCTATTCTGGTTATAACAAA)-3′. The amplified DNA containing 3′UTR sequence was cloned into vector pcDNA3.1(+) by restriction enzyme sites, *Hin*dIII and *Eco*RI.

### Western blot analysis

Polyvinylidene difluoride (PVDF) membranes were blocked with Tris-buffered saline/0.1% (vol/vol) Tween 20 containing 5% non-fat dry milk and probed with the indicated antibodies. Antibodies against Dicer (sc-136981; Santa Cruz), Drosha (ab12286, Abcam), La protein (sc-33593, Santa Cruz), PTB (sc-16547, Santa Cruz), HuR (39–0600, Invitrogen), KSRP / FBP2 (A302–021A, Bethyl laboratories), hnRNPA1 (GTX106208, GenTex), FLAG (SIGMA) and actin (Chemicon) were used. After washing, the membranes were incubated with an HRP-conjugated anti-mouse antibody or HRP-conjugated anti-rabbit antibody, as appropriate (diluted 1:5000). HRP was detected using the Western Lightning Chemiluminescence Kit, following the manufacturer instructions (GE Healthcare).

### Immunoprecipitation of FLAG-Dicer proteins

The SF268 cells grown in each well of a 6-well plate were transfected with 5 μg of plasmids FLAG-Dicer, FLAG-Dicer-MUT, or FLAG-Dicer-dsRBD by 5 μl of Lipofectamine 2000 (Invitrogen). After 48 h post-transfection, the transfected cells were lysed by incubation with a lysis buffer (500 mM NaCl, 20 mM Tris-Cl, pH 8.0, 1 mM EDTA, 1% Triton X-100) on ice for 30 min and then subjected to a vortex for 30 s at room temperature. The cell lysates were harvested from the supernatant of the lysed cells after centrifugation at 16,000 *g* for 10 min. Two milligrams of cell lysates from the transfected cells were incubated with 40 μl of the resin conjugated with anti-FLAG M2 antibody (Sigma) at 4°C for 2 h. The immunoprecipitated FLAG-Dicer proteins with resin were washed in a lysis buffer four times and then washed with Buffer D (200 mM KCl, 20 mM Tris-Cl pH 8.0, 20 mM EDTA) three times. The resin with FLAG-Dicer proteins was then resuspended in 60 μl of a resuspension buffer (40 mM Tris-Cl pH 7.5, 500 mM NaCl, 5 mM MgCl2, 40% glycerol) and stored in a freezer at –80°C.

### In vitro transcription

To generate EV71-IRES-FLuc reporter RNA and Polio-IRES-FLuc reporter RNA, plasmid pGL3-EV71-5′-UTR-Fluc and pGL3-Polio-5′-UTR-Flu were linearized by *Xho*I to be used as the template for in vitro transcription. To generate cap-RLuc reporter RNA, the DNA fragment containing the T7 promoter and Renilla gene were amplified from the pRH plasmids (26) by using primers [5′-(TAATACGACTCACTATAGGCTAGCCACCATGACTTCGAAAGTTTATGATC)-3′] and [5′-(TTATTGTTCATTTTTGAGAACT)-3′] as the template for in vitro transcription. The cap structure on the 5′-end of the Renilla reporter RNA was added by the Vaccinia Capping System (New England BioLabs). To generate EV71 5′ UTR RNA, a T7 promoter-EV71-5′ UTR fragment flanked by *Eco*RI site was excised from the plasmid pT7-EV71-IRES to form a template for in vitro transcription. To generate EV71 3′UTR RNA, a T7 promoter-EV71–3′UTR fragment flanked by *Eco*RI site was excised from the plasmid pT7-EV71–3′UTR to form a template for in vitro transcription. To generate EV71 replicon RNA, the EV71 replicon plasmid was linearized by *Sal*I for producing the DNA template for in vitro transcription. RNA transcripts were synthesized using a MEGAscript T7 kit (Ambion), following the manufacturer instructions. To generate sponge RNA against vsRNA1 or control-sponge RNA, the sponge-vsRNA1 plasmid/sponge-control plasmid was linearized by *Eco*RI/*Xho*I. The linearized DNA was used as template for in vitro transcription.

Biotinylated EV71 5′ UTR RNA was synthesized in a 20 μl MEGAscript transcription reaction mixture by adding 1.25 μl of 20 μM of Bio-16-UTP, a biotinylated UTP that can replace UTP in the in vitro transcription for RNA labelling (Roche). The ^32^P-labelled EV71 5′ UTR RNA was synthesized in a 20 μl MEGAscript transcription reaction mixture by adding α-^32^P-UTP. Synthesized RNAs were purified using the RNeasy Protect Mini Kit (Qiagen).

### IRES-FLuc and Cap-RLuc reporter assay

To assay the effects of vsRNA1 on EV71 IRES or Poliovirus IRES activities, SF268 cells were cotransfected with either 160 pmol of vsRNA1 mimic (5′-GUAACUUAGAAGCUGUAAAUCAACGAUCA-3′) or scrambled vsRNA1 (5′-AAUGCUAUGAGACUAAUGAUACCAAGACU-3′) concurrently with EV71 IRES FLuc or Polio IRES FLuc or cap-RLuc reporter RNAs. After 6 h of reporter RNA transfection, cell extracts from the transfected cells were prepared in Cell Culture Lysis Reagent (Promega). The firefly luciferase activity of the cell extracts was assayed using the Luciferase assay system (Promega), following the manufacturer instructions. The renilla luciferase activity of the cell extracts was assayed using the Dual luciferase reporter assay system (Promega).

### Detection of IFN-β mRNA in RNAs-treated cells

RNA from 10^6^ of SF268 cells treated with 2 μg of Lipofectamine 2000, transfected with 320 pmol of vsRNA1 or scrambled vsRNA1 or 2 μg of poly (I:C) (Sigma) for 24 h were isolated using TRIzol reagent (Sigma). Two μg of the RNA from each sample was taken as template to generate complementary DNA (cDNA) by reverse transcription using oligo(dT) primer. To detect the IFN-β cDNA by RT-PCR, a set of primers, forward 5′-(AGAAGGAGGACGCCGCATTG)-3′ and reverse 5′-(TCAGTTTCGGAGGTAACCTG)-3′ were used. The cDNA of β-actin was amplified using primers 5′-(CTACAATGAGCTGCGTGTGG)-3′ and 5′-(GCTCATTGCCAATGGTGATG)- 3′. The amplified DNA was then submitted to agarose gel electrophoresis. The detection of IFN-β mRNA by real-time quantitative PCR was performed as previously described (35). The results were normalized to the levels of the housekeeping gene β-actin using the 2-^ΔΔC^_T_ method.

### In vitro Dicer cleavage assay of EV71 5′UTR and replicon RNA

To assay recombinant Dicer cleaving EV71 5′ UTR RNA, 10,000 cpm of ^32^P-labelled EV71 5′ UTR RNA was incubated with various amounts (from 0.05 to 0. 25 U) of commercial recombinant Dicer enzyme (T510002, Genlantis) and cleavage buffer (20 mM Tris-Cl pH 7.5, 250 mM NaCl, 2.5 mM MgCl2 and 0.1 μg/μl bacteria tRNA) at an overall volume of 10 μl. The mixture was incubated at 37°C for 1.5 h. After incubation, the reactants were mixed with 10 ml of loading dye (80% formamide, 1 mM EDTA, 0.1% bromophenol blue and 0.1% Xylene Cyanol) and assayed using denaturing 3.5% polyacrylamide gel (1X TBE, 8M Urea, acrylamide [19:1; acrylamide:bisacrylamide]). For generating svRNA by Dicer-treated EV71 5′ UTR, 10 μg of EV71 5′ UTR RNA was treated with 1 U of a recombinant dicer enzyme (T510002, Genlantis), following the manufacturer instructions. The Dicer-treated RNAs were assayed by northern blotting. To determine the catalytic activity of FLAG-Dicer proteins, each reaction involved 10 μl of immunoprecipitated FLAG-Dicer proteins with resin. Next, 400,000 cpm samples of ^32^P EV71 5′ UTR RNAs were incubated with an immunoprecipitated FLAG-Dicer, FLAG-Dicer-MUT, or FLAG-Dicer-dsRBD proteins, cleavage buffer and an additional 4 U/μl of RNaseOUT recombinant ribonuclease inhibitor (Invitrogen) in a volume of 10 μl. The mixtures were incubated at 37°C for 1.5 h. After incubation, the treated RNAs were isolated from the reactants by the TRIzol protocol (Invitrogen) and assayed by denaturing 3.5% polyacrylamide gel (1X TBE, 8M Urea, acrylamide [19:1; acrylamide:bisacrylamide]).

### Pull-down assay for biotinylated EV71 5′UTR RNA with streptavidin beads

Three micrograms of biotinylated EV71 5′ UTR RNA and 200 μg of cell extracts from SF268 (prepared as described previously (28)]) were added to the RNA interaction buffer (30 mM Tris-HCl pH 6.8, 3 mM MgCl_2_, 50 mM NaCl, 0.1% Triton X-100, 20% glycerol, 1 mM DTT and 2 mM EDTA) in a reaction volume of 100 μl. The reaction mixture was incubated at 30°C for 15 min. The Streptavidin MagneSphere Paramagnetic Particles (Promega) were washed with a 0.5× SSC buffer (150 mM NaCl and 15 mM trisodium citrate dihydrate) three times and were then added to the reaction mixture for 10 min at room temperature. For the competition assay, a poly(A) RNA was used (Sigma). The pull-down protein-RNA complex was washed in 0.1× SSC five times. After washing, the beads with the pull-down protein-RNA complex were incubated with an SDS–PAGE sample buffer at 25°C for 10 min and then centrifuged at 21,000 *g* for 10 min. The supernatant containing the pull-down proteins was then assayed by western blotting.

### Native gel analysis of the Dicer-EV71 5′UTR complex

Recombinant Dicer Enzyme (T510002, Genlantis) was incubated in 20 mM Tris-HCl pH 7.5, 50 mM KCl, 5 mM MgCl_2_, 1 mM DTT, and 5% RNaseOUT for 10 min at room temperature at a volume of 10 μl, after which 18,000 cpm of ^32^P-labelled EV71 5′ UTR RNA was added to the Dicer reaction for an overall volume of 15 μl, and was cooled on ice for 30 min. To study the competition by cold EV71 5′UTR RNA, unlabelled EV71 5′ UTR RNA and ^32^P-labelled EV71 5′ UTR RNA were added to the reaction simultaneously. After incubation, a 1.5 μl loading buffer (60% glycerol, 0.2% bromophenol blue) was added to the reaction. The RNA-protein complex was assayed by non-denaturing 3% polyacrylamide TBE gels (29:1; acrylamide:bisacrylamide) and was detected by autoradiography.

### Fluorescence microscopy

RD cells grown on glass cover slips were infected with EV71/4643/MP4 at a MOI of 40. At 6 h p.i., the infected cells were washed 3x with phosphate buffered saline (PBS). The cells on the cover slips were fixed with 4% (wt/vol) paraformaldehyde at room temperature for 20 min. After washing 3x with PBS, the cells on the cover slips were permeabilized in 0.3% Triton X-100 at room temperature for 5 min and washed again with PBS. For Dicer and viral 2B immunostaining, the samples were blocked in solution [PBS, containing 5% bovine serum albumin (BSA)] for 60 min at 37°C and then incubated with an anti-Dicer antibody (sc-136981, Santa Cruz) and polyclonal anti-2B antibody (1:200) for 2 h at 37°C. After washing with PBS for three times, the samples were reacted with Alexa Fluor 488-conjugated goat anti-mouse IgG (Life technologies) and Alexa Fluor 568-conjugated goat anti-rat IgG (Life technologies) at 37°C for 1.5 h. For Drosha and viral 3D immunostaining, the samples were blocked in solution (PBS, containing 5% bovine serum albumin (BSA)) for 60 min at 37°C and then incubated with an anti-Drosha antibody (1:25) (ab12286, Abcam) and monoclonal anti-3D antibody (1:200) for 2 h at 37°C. After washing 3x with PBS, the samples were incubated with Alexa Fluor 488-conjugated goat anti-rabbit IgG and Alexa Fluor 594-conjugated goat anti-mouse IgG (Life technologies) at 37°C for 1.5 h. After washing with PBS, the samples were treated with Hoechst 33258 for 15 min at room temperature and washed with PBS three more times. Finally, the cover slips with adhering cells were placed on a glass slide and sealed with transparent nail polish. Images were captured by confocal laser scanning microscopy (ZEISS LSM510 META).

### Viral growth in cells transfected with anti-vsRNA1 sponge RNA and vsRNA1

RD cells were infected with EV71/4643/MP4 at an MOI of 10. The virus was adsorbed for 1 h at 37°C. After viral adsorption, the infected cells in each well were transfected with 160 pmol of synthetic mimic vsRNA1 (5′-GUAACUUAGAAGCUGUAAAUCAACGAUCA-3′) or scrambled vsRNA1 (5′-AAUGCUAUGAGACUAAUGAUACCAAGACU-3′) and 4 μl of Lipofectamine 2000 (Invitrogen). For the anti-vsRNA1 sponge RNA experiments, the infected cells were transfected with 1 μg of the anti-vsRNA1 sponge or scrambled RNA and 2 μl of Lipofectamine 2000 (Invitrogen). For the LNA experiment, the infected cells were transfected with 80 pmol/ml of LNA-vsRNA1 (5′ LNA-TUGAUUUACAGCUUCU) or LNA-control (5′ LNA-CAGUACUUUUGUGUAGUACAA) after virus adsorption. The viral titer was determined using plaque assays with RD cells.

### ^35^S-methionine/cysteine labelling

We seeded 2.5 × 10^5^ RD cells into each well of 12-well plates and incubated them for 24 h. The cells were then infected with EV71/4643/MP4 at an MOI of 10. The virus was adsorbed for 1 h at 37°C. After virus adsorption, the infected cells in each well were transfected with 160 pmol of synthetic mimic vsRNA1 (5′-GUAACUUAGAAGCUGUAAAUCAACGAUCA-3′) or scrambled vsRNA1 (5′- AAUGCUAUGAGACUAAUGAUACCAAGACU -3′) by 4 μl of Lipofectamine 2000 (Invitrogen). For anti-vsRNA1 sponge experiments, the infected cells were transfected with 1 μg of sponge RNA or control RNA. The medium was then replaced with methionine/cysteine-free DMEM, and incubated again at 37°C. At 4 h p.i., the medium was replaced with another medium containing ^35^S-Met labelling (50 mCi/ml). After 1 h of labelling, the cell monolayers were washed with PBS and lysed with a lysis buffer. Cell lysates were isolated by centrifugation at 12,000 *g* for 10 min at 4°C, and the supernatants were collected for further analysis. Radiolabelled proteins were resolved by SDS–PAGE (12% gels), transferred to a PVDF membrane, and detected by autoradiography.

### Preparation of SF268 cell extracts for in vitro translation assay

To perform the in vitro IRES activity assay, SF268 cells were washed in PBS and removed by scraping. The packed cells were resuspended in a hypotonic buffer (10 mM HEPES pH 7.9, 1.5 mM MgCl_2_, 10 mM KCl and 0.5 mM DTT) and homogenized using a 25G needle. Homogenized cell fragments were mixed with one volume of a working buffer (40 mM HEPES, 40% glycerol, 200 mM KCl, 0.4 mM EDTA and 1 mM DTT at pH = 7.4) and vortexed for 30 s. After centrifugation at 25,000 *g*, the extracted cell proteins in the supernatant were harvested and stored at –80°C.

### In vitro translation assay

The study co-incubated 0.5 μg of EV71 5′ UTR FLuc reporter RNA and 60 pmol of vsRNA1 or scrambled vsRNA1 or modified vsRNA1 containing vsRNA1_163_–_165_ [5′-(GUAACUUAGAAGCUGUAA**A**AGU**A**CGAUCA)-3′], vsRNA1_174_–_176_ [5′-(GUAACUUACUUGCUGUAAAUCAACGAUCA)-3′] and vsRNA1_163_–_165/174–176_ [5′-(GUAACUUACUUGCUGUAAAAGUACGAUCA)-3′] with 50 μg SF268 cell extracts and 4U RNaseOUT recombinant ribonuclease inhibitor (Invitrogen) in a volume of 20 μl. The mixtures were incubated at 30°C for 5 min. After incubation, the reactions were used to perform in vitro translation at a volume of 25 μl. These mixtures contained 0.5 μg EV71 5′ UTR FLuc reporter RNA, 60 pmol of mimic RNA1 or scrambled vsRNA1, 50 μg of SF268 cell extract and 20% rabbit reticulocyte lysate (RRL) (Promega). The in vitro IRES activity assay followed the protocol of the Rabbit Reticulocyte Lysate System (Promega). The mixtures were incubated at 30°C for 90 min, and firefly and Renilla luciferase activity was measured using the Luciferase assay system (Promega).

### Polysome profiling

7.2 × 10^6^ SF268 cells were infected with EV71 at a MOI of 10. After 1 h adsorption, cells were transfected with the control (scrambled) RNA or vsRNA1 mimic for 6 h. Cells were treated with 0.1 mg/ml cycloheximide for 5 min at 37°C, and then washed twice with ice-cold PBS containing 0.1 mg/ml cycloheximide. Cells were then lysed using 1 ml polysomal extraction buffer (20 mM Tris-Cl, pH 7.5, 5 mM MgCl_2_, 100 mM KCl, 1% Triton-X-100 and 0.1 mg/ml cycloheximide) on ice for 30 min. Cell debris was removed by centrifugation at 15,000 *g* for 5 min, and the cell extracts were layered onto 10 ml 7–47% sucrose gradients (comprising 20 mM Tris-Cl pH 7.5, 5 mM MgCl_2_ and 100 mM KCl). Subsequently, the gradients were centrifuged at 35,000 rpm for 150 min in a Beckman SW41-Ti rotor at 4°C. The gradients were fractionated with the Isco fractionator by pumping a 60% sucrose solution into the bottom of the tube. The fractions were then collected from the top of the tube and the optical density was measured at 254 nm (OD254). The ribosome subunits were detected in each fraction by Western blotting using specific antibodies directed against the S6 (#2211; Cell Signaling) and P0 (NBP1–57528; Novus) ribosomal proteins.

### Statistical analysis

Significant differences were determined by performing two-tailed Student's *t*-test using Prism 5 software (GraphPad).

## RESULTS

### EV71 infection generates virus-derived small RNAs

**Figure 1.**
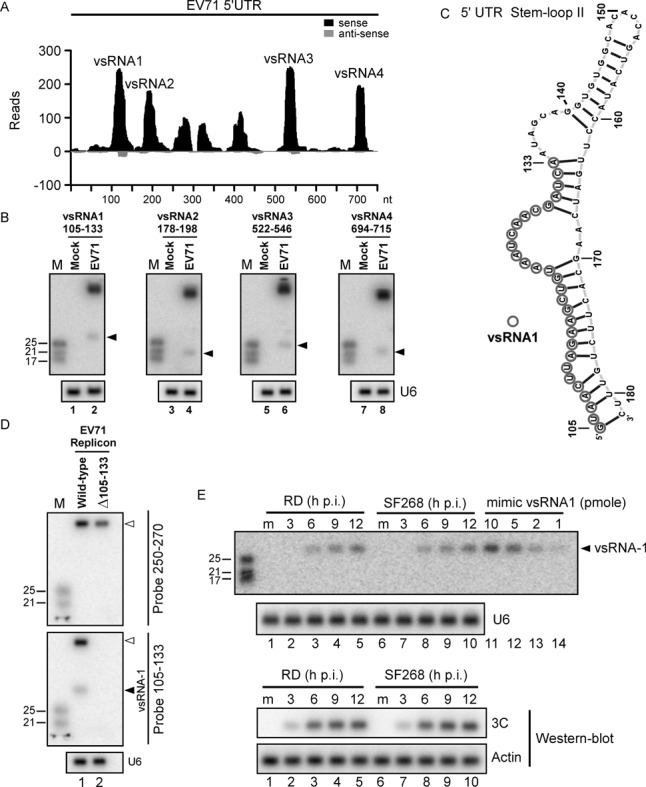
Identification of vsRNAs within the EV71 5′UTR. (**A**) Virus-derived small RNAs (vsRNAs) in SF268 cells infected with EV71 were sequenced using Illumina technology. The position distributions and abundance (reads) of that sequenced vsRNAs that perfectly matched the EV71 5′UTR are shown. (**B**) vsRNA1–4 (black arrow) in mock- or EV71-infected SF268 cells were detected with ^32^P-labelled probes containing nucleotides against positions 105–133, 178–198, 522–546 and 694–715 of the EV71 5′UTR. U6 snRNA was used as a loading control. (**C**) The secondary RNA structure of stem-loop II of the EV71 5′UTR was predicted using Context Fold software (37) and was illustrated with jViz.RNA 2.0 software (http://jviz.cs.sfu.ca/). Circles indicate the predicted vsRNA1 generation site, based on the deep sequencing result. (**D**) Precursor RNA (upper panel) and vsRNA1 (middle panel) from wild-type EV71 replicon or the vsRNA1 deletion mutant (Δ105–133) replicon EV71 in transfected SF268 cells were detected using ^32^P-labelled probes against nt 250–270 and nt 105–133 of the viral genome. U6 snRNA was used as a loading control (lower panel). (**E**) At 3, 6, 9 and 12 h p.i. with EV71, vsRNA1 in human rhabdomyosarcoma (RD) and SF268 cells (from 2.5 × 10^6^ cells per lane) was detected and compared to the indicated levels of synthetic vsRNA1 (mimic vsRNA1). RNA in mock-infected cells (m) served as a negative control. Viral protein 3C and actin were detected by western blotting.

Previous studies have shown that the insertion of a heterologous miRNA-precursor stem-loop sequence element into a cytoplasmic RNA virus can induce the production of vsRNA in infected mammalian cells (11–13). Moreover, a subset of positive-stranded cytoplasmic RNA viruses contain a highly structured IRES region in their genomic RNA (36). The 5′UTR of the EV71 genome (nt 1–745) contains a highly structured IRES and a cloverleaf, which is associated with viral replication and translation (19). To determine whether vsRNA was generated from the 5′UTR during EV71 infection, we deep sequenced small RNAs under 50 nt in EV71-infected SF268 cells. Only 0.05% of the detected total RNA (17 922 of 33 539 716 reads) was derived from viral RNA, which is consistent with findings by Parameswaran *et al.* (14). Among these vsRNAs, most of the reads (98%) were derived from viral positive-stranded RNA. Small RNAs in infected cells containing sequences that perfectly matched the 5′UTR of viral genomic RNA were identified (Figure 1A). We named these vsRNAs vsRNA1, vsRNA2, vsRNA3 and vsRNA4. Three of the same small RNAs, vsRNA1, vsRNA2 and vsRNA3, were also detected in EV71-infected RD cells (Supplementary Figure S1), suggesting that these small RNAs are generated by a specific mechanism rather than by random degradation.

**Figure 2.**
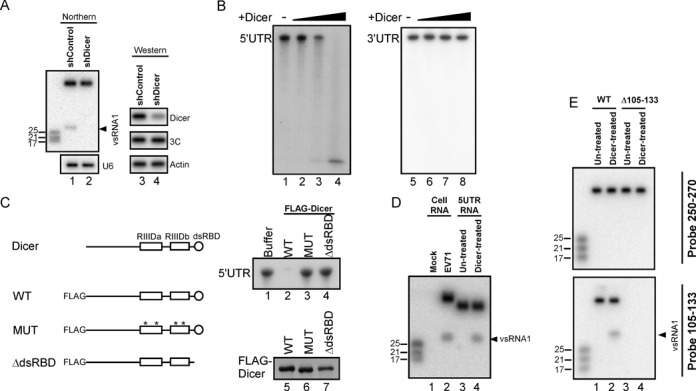
Dicer cleaves the EV71 5′UTR into vsRNA1. (**A**) vsRNA1 in SF268 cells transfected with the control plasmid (shControl) or a plasmid expressing shRNA against Dicer (shDicer) was detected using the vsRNA1 probe. The levels of Dicer and viral 3C protein in these cells were detected by western blotting. (**B**) In vitro EV71 5′UTR cleavage assay with recombinant Dicer. The ^32^P -labelled EV71 5′UTR and 3′UTR RNA was detected after being incubated with reaction buffer only (-) or with various amounts (from 0.005 to 0.25 U) of recombinant Dicer for 1.5 h at 37°C. (**C**) Dicer protein contains 2 RNAse III domains (RIIIDa and RIIIDb) and a dsRBD. N-terminal FLAG-fused Dicer protein (WT) and mutated Dicer proteins with 4 point mutations (*) in the catalytic sites of RIIIDa / RIIIDb (MUT) and with a dsRBD deletion (ΔdsRBD) were used for the in vitro cleavage of the EV71 5′UTR RNA. The ^32^P-labelled EV71 5′UTR RNA was detected after being incubated with reaction buffer or with these FLAG-Dicer proteins at 37°C for 1.5 h (upper right panel). The FLAG-Dicer proteins in each reaction were detected using an antibody against the FLAG peptide (lower right panel). (**D**) A vsRNA1 probe was used to detect vsRNA1 generated by EV71-infected cells (Cell RNA) and by synthetic EV71 5′UTR RNA after Dicer treatment (Dicer-treated). RNA isolated from mock-infected cells and from a synthetic 5′UTR without Dicer treatment (Un-treated) served as negative controls. (**E**) Detection of vsRNA1 in wild-type (WT) or Δ105–133 mutant EV71 replicon RNA treated with recombinant Dicer. Full-length replicon RNA and vsRNA1 were detected using a ^32^P-labelled probe against nt 250–270 and nt 105–133 of the viral genome.

To confirm the existence of vsRNAs in EV71-infected cells, we designed probes based on the most widespread vsRNA sequences in the deep-sequencing results (Figure 1A). Northern blots showed that vsRNAs 1, 2, 3 and 4 were generated in infected SF268 cells (Figure 1B). These results also suggested that only a small portion of the precursor was processed into vsRNA, which was similar to previous findings in another RNA virus (8). Among the four vsRNAs detected in the 5′UTR of SF268 cells, the dominant vsRNA (i.e., vsRNA1) showing the highest number of reads was selected for further study. Based on the deep sequencing results (Figure 1A), the most widespread coverage of vsRNA1 was derived from nt 105–133 of the EV71 5′UTR, which is located within the double-stranded region of the predicted stem-loop II structure, illustrated by Context Fold software (37) (Figure 1C). To confirm whether vsRNA1 was generated from nt 105–133 of the EV71 5′UTR, we generated an EV71 replicon in which nt 105–133 of the EV71 5′UTR were deleted (Rep-Δ105–133). Only wild-type (WT) EV71 replicon-transfected cells generated vsRNA1 (Figure 1D, lower panel). Taken together with the in vitro cleavage data (see below; Figure 2E), this result confirmed that the vsRNA1 generation site is located in nt 105–133. A single cell must have a minimum of 100 copies of miRNA to be capable of specific functions (13,38). To elucidate whether EV71 infection could generate a sufficient amount of vsRNA1, we quantified the levels of vsRNA1 in 2.5 × 10^6^ infected cells by comparing them with known amounts of synthetic vsRNA1 (Figure 1E). Approximately 3.6 × 10^5^, 7.2 × 10^5^ and 1.2 × 10^6^ copies of vsRNA1 were produced in a single infected cell at 6, 9 and 12 h p.i. (1.5 pmol [6 h p.i.], 3 pmol [9 h p.i.] and 5 pmol [12 h p.i.] of vsRNA1 in 2.5 × 10^6^ cells). Argonaute2 (Ago2) associates with miRNA as functional partner to induce targeted RNA degeneration or translational repression (39–41). To examine whether vsRNA1 associated with Ago2, we performed an immunoprecipitation assay. An anti-Ago2 antibody enriched both vsRNA1 and an endogenous miRNA (miR-21) from infected cells (Supplementary Figure S2). In summary, we found that EV71 infection generated a substantial level of vsRNA1, which associated with Ago2 in infected cells.

### Dicer cleaves EV71 5′UTR and generates vsRNA1

The previous result demonstrated that EV71-infected cells could generate a substantial level of vsRNA1 (Figure 1). We were curious about how vsRNA1 is generated in infected cells. The canonical mechanism of the production of small RNAs or miRNAs in mammalian cells requires both nuclear and cytoplasmic activities (1). However, EV71 is a cytoplasmic virus; it cannot access the nucleus for vsRNA generation. A non-canonical, Dicer-dependent but DGCR8-independent pathway has been shown to generate functional small RNA from a cytoplasmic RNA virus (12,13). The Dicer enzyme cleaves structured RNAs into various small RNAs (7,42–43). Therefore, we hypothesised that Dicer processes stem-loop II of the EV71 5′UTR (Figure 1C) into vsRNA1 in virus-infected cells. To investigate whether Dicer is involved in the generation of vsRNA1, we attempted to detect vsRNA1 in Dicer-depleted cells infected with EV71. Unlike the measurable signal in control cells (Figure 2A, lane 1), vsRNA1 was undetectable in the Dicer-depleted cells (lane 2). This result suggested that Dicer is involved in vsRNA1 generation during EV71 infection. Moreover, the same experiments demonstrated that the generations of vsRNA2, vsRNA3 and vsRNA4 are also Dicer-dependent (Supplementary Figure S3). Ago2 is involved in non-canonical cytoplasmic small RNA processing (44,45). To verify that Ago2 participates in vsRNA1 generation, we quantified vsRNA1 in Ago2-depleted cells infected with EV71; Ago2 knockdown did not affect vsRNA1 generation (Supplementary Figure S4). An in vitro cleavage assay was performed to determine whether Dicer cleaves the EV71 5′UTR. An increase in the amount of recombinant Dicer resulted in a reduction of the full-length ^32^P-labelled EV71 5′UTR (Figure 2B, lanes 1–4) without affecting EV71 3′UTR (lanes 5–8), indicating that Dicer specifically cleaves viral RNAs at this region. As Figure 2C shows, Dicer contains 2 RNase III catalytic domains (RIIIDa and RIIIDb) and a double-stranded RNA-binding domain (dsRBD), which are essential for Dicer cleavage (31). We further confirmed the potential Dicer cleavage on 5′UTR using 2 Dicer mutants as controls: Dicer-MUT (mutations in the catalytic sites of the two RNase III domains abrogate the RNase activity (46)) and Dicer-ΔdsRBD (a deletion mutant of dsRBD). Dicer-WT, Dicer-MUT and Dicer-ΔdsRBD were fused to FLAG and transfected into SF268 cells. FLAG-immunoprecipitation (IP) was performed to purify Dicer proteins, which were then quantified (Figure 2C, lower right panel). The WT and mutant forms of Dicer were used for in vitro cleavage assays. Dicer-WT (Figure 2C, upper right panel, lane 2), but not Dicer-MUT or Dicer-ΔdsRBD (lanes 3 and 4), caused a reduction in the level of EV71 5′UTR RNA, suggesting that Dicer may cleave viral RNA through its RNase activity. Recombinant Dicer was also noticed to cleave RNAs other than EV71 5′UTR in vitro, which may be a result of RNase activity. To confirm that Dicer specifically generated vsRNA1 in infected cells after cleaving the EV71 5′UTR RNA, we compared the vsRNA1 produced in virus-infected cells to that produced in the in vitro assays (5′UTR RNA + Dicer). Both EV71 infection and the Dicer-treated 5′UTR RNA generated vsRNA1 (Figure 2D). Because Dicer can cleave double-stranded RNA into RNA duplexes, we also sought to confirm whether Dicer cleavage may produce small RNA from 163–181 nt, the complement region of the vsRNA1-generation site in SLII (Figure 1C). The results indicated that both EV71 infection and Dicer-treated 5′UTR RNA can generate small RNA 163–181 (Supplementary Figure S5). We previously demonstrated that vsRNA1 was not generated in cells transfected with the EV71 Δ105–133 replicon (Figure 1D). To further confirm that Dicer generated vsRNA1 from the same region of viral RNA as in infected cells, we treated the WT and mutant (Rep-Δ105–133) replicon RNA with Dicer and examined the generation of vsRNA1. vsRNA1 was detected in the Dicer-treated WT replicon (Figure 2E, lower panel, lane 2) but not in the Dicer-treated Rep-Δ105–133 (lane 4). No nonspecific small RNA fragments were detected by probing regions other than vsRNA1 (Figure 2E, upper panel), suggesting that the in vitro Dicer cleavage and the viral infection generated vsRNA1 from the same region of the viral RNA (Figures 1D and 2E). Overall, these results suggest that Dicer cleaves the EV71 5′UTR and generates vsRNA1 from nt 105–133 of the EV71 5′UTR in infected cells.

### The association of Dicer and EV71 5′UTR RNA

**Figure 3.**
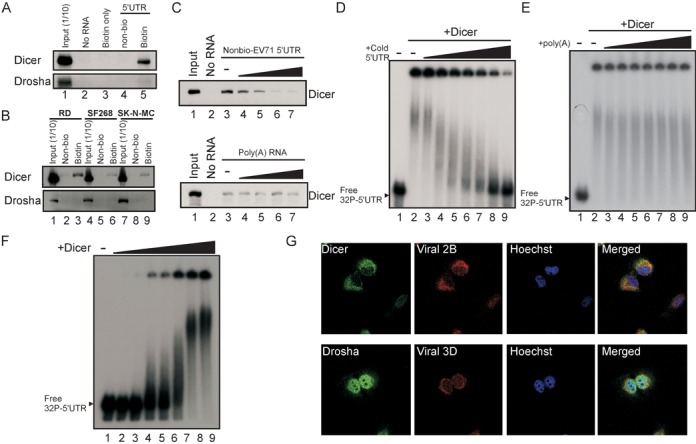
The association between Dicer and the EV71 IRES. (**A**) In vitro Dicer and Drosha were coprecipitated with EV71 5′UTR. Proteins from SF268 cell lysates were incubated with biotin-UTP (biotin only), unlabelled (Non-bio), or biotin-labelled (Biotin) EV71 5′UTR RNA and streptavidin beads. Proteins from lysates without incubation (No RNA) were pulled down by the same beads and served as a negative control. The Dicer and Drosha proteins in the pull-down reactions or in 1/10 of the SF268 lysate before the pull-down (Input) were detected using western blotting. (**B**) The same in vitro Dicer and Drosha pull-down assay as (A) was performed on lysates from RD, SK-N-MC and SF268 cells. (**C**) Competition assay for Dicer association with the EV71 5′UTR. Lane 1 contained the cell lysate (Input) only. The unlabelled EV71 5′UTR (Nonbio-EV71 5′UTR; zero (-) to 30 μg) or poly(A) RNA was added to compete with 3 μg of the biotinylated EV71 5′UTR probe interacting with Dicer in the SF268 cell lysate. Protein that was pulled down without adding any RNA was the negative control (No RNA). (**D** and **E**) The complex between recombinant Dicer and the EV71 5′UTR RNA. The ^32^P-labelled EV71 5′UTR RNA was incubated with 0.4 U of recombinant Dicer (+Dicer) and varying amounts (40 to 400 ng) of unlabelled EV71 5′UTR RNA (+cold 5′UTR) (D) or poly(A) RNA (E). The resulting complexes were analysed using native gel [non-denaturing 3% polyacrylamide TBE gels (29:1 acryamide:bisacryamide)] ectrophoresis. EV71 5′UTR RNA that was not incubated with Dicer and cold 5′UTR (Free ^32^P 5′UTR) were used to determine the free RNA size. (**F**) EV71 5′UTR RNA-protein complex formed with varying amounts of recombinant Dicer. Free ^32^P-labelled EV71 5′UTR RNA only (-) or 5′UTR RNA incubated with varying amounts (0.08 to 0.8 U) of recombinant Dicer (+Dicer) was analysed using the same native gel electrophoresis. (**G**) The locations of Dicer and Drosha in EV71-infected SF268 cells were detected using specific antibodies at 6 h p.i. The viral 2B and 3D proteins in SF268 cells were used as markers of infection. The nuclei of the cells were stained with Hoechst dye.

The binding of Dicer to double-stranded RNA is crucial for the recognition and cleavage of Dicer's substrate (31,47–48). Because Dicer dsRBD is also essential for EV71 5′UTR cleavage (Figure 2C, upper right panel), we examined whether Dicer specifically associated with the EV71 5′UTR RNA. Dicer RNase III activity requires Mg^2+^, and the RNase activity of Dicer was found to be inhibited by EDTA in a Dicer-binding assay (46). Here, we used EDTA in an RNA pull-down assay to examine whether endogenous Dicer associates with the viral 5′UTR. A biotinylated RNA containing the EV71 5′UTR was incubated with an SF268 cell lysate, and streptavidin beads were used to pull down biotinylated RNA and its associated proteins. Using western blotting, we detected Dicer in the RNA-protein complex (Figure 3A, lane 5). Drosha, another RNase III with a significant role in cellular miRNA biogenesis, was not present in the complex (lower panel, lane 5). Dicer was associated with the EV71 5′UTR not only in SF268 (human glioblastoma) cells but also in RD (human rhabdomyosarcoma) and SK-N-MC (human neuroepithelioma) cells (Figure 3B, lanes 3, 6 and 9). This association was confirmed by a competition assay with unlabelled EV71 5′UTR RNA (Figure 3C, upper panel) or unstructured poly(A) RNA (lower panel). Native gel electrophoresis confirmed the interaction between Dicer and the 5′UTR. The ^32^P-labelled 5′UTR (745 nt) formed a complex with recombinant Dicer, and the migration of the RNA-protein complex was retarded in the native gel (Figure 3D, lane 2). The retardation decreased and the free RNA increased after cold competitor RNA (EV71 5′UTR) was added (Figure 3D, lanes 3–9). There was no effect on the 5′UTR-Dicer complex when nonspecific poly(A) RNA was added to the reactions (Figure 3E, lane 2 versus lanes 3–9). The RNA binding of Dicer was dose-dependent (Figure 3F). These results indicated that Dicer associates with the EV71 5′UTR in vitro. Next, we examined the location of Dicer in EV71-infected cells. EV71 replicates in the cytoplasm, whereas the localisation of several cellular proteins is altered in virus-infected cells (26–27,29,49). A recent report also showed that Drosha relocalised to the cytoplasm and contributed to viral RNA processing in Sindbis virus-infected cells (13). Therefore, we examined the locations of both Dicer and Drosha in EV71-infected cells. The results showed that the majority of Dicer was distributed in the cytoplasm (Figure 3G, upper panel), whereas Drosha was found mainly in the nucleus (lower panel). These results further supported the conclusion that Dicer cleaves the EV71 5′UTR to generate vsRNA1.

### vsRNA1 downregulates virus infection and viral protein synthesis

**Figure 4.**
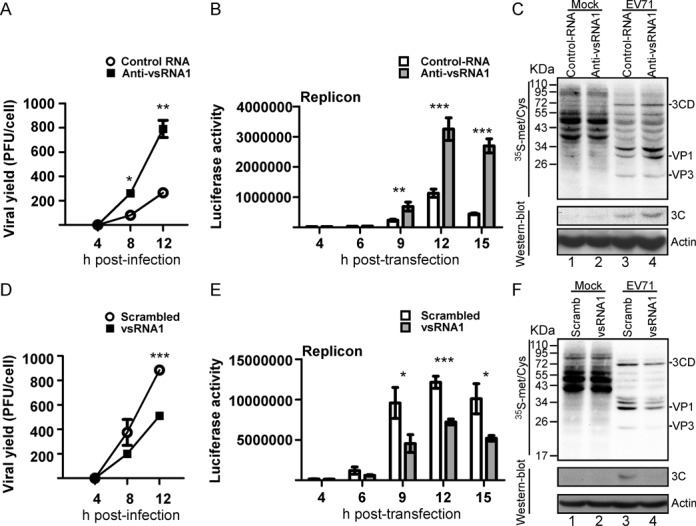
The effect of vsRNA1 on virus infection and viral protein synthesis. (**A**) Effect of anti-vsRNA1 sponge RNA on EV71 viral growth. EV71-infected RD cells were transfected with the anti-vsRNA1 sponge or control RNA. After viral adsorption, viruses from the debris and the supernatant were collected at 4, 8 and 12 h p.i. The viral yields were determined with plaque assays. Error bars represent standard deviations for duplicate assays (*P < 0.05; **P < 0.01, Student's *t*-test). (**B**) Effect of anti-vsRNA1 sponge RNA on EV71 replication. The luciferase activity in the EV71 replicon in RD cells cotransfected with anti-vsRNA1 sponge or control RNA was monitored at 4, 6, 9, 12 and 15 h post-transfection. Error bars represent standard deviations for three independent experiments (*P < 0.01; ***P < 0.001; Student's *t*-test). (**C**) Effects of anti-vsRNA1 sponge on viral and host protein synthesis. Mock- or EV71-infected cells were transfected with the anti-vsRNA1 sponge or control RNA. Protein synthesis in these cells was examined using ^35^S-methionine/cysteine-labelling between 6 and 7 h p.i. The labelled viral proteins were identified according to their sizes. Viral protein 3C in the cell lysates was detected by western blotting. (**D**) Effect of vsRNA1 on EV71 viral growth. EV71-infected RD cells were transfected with vsRNA1 mimic or scrambled RNA. After viral adsorption, viruses from the debris and the supernatant were collected at 4, 8 and 12 h p.i. The viral yields were determined using plaque assays. Error bars represent standard deviations for duplicate assays (***P <0.001), Student's *t*-test). (**E**) Effect of the vsRNA1 mimic on EV71 replication. Luciferase activity in the EV71 replicon in RD cells cotransfected with vsRNA1 mimic or scrambled RNA was monitored at 4, 6, 9, 12 and 15 h post-transfection. Error bars represent standard deviations for three independent experiments (*P < 0.05, ***P < 0.001, Student's *t*-test). (**F**) Effects of vsRNA1 on viral and host protein synthesis. Mock- or EV71-infected cells were transfected with vsRNA1 mimic or scrambled RNA (Scramb). Protein synthesis in these cells was examined using ^35^S-methionine/cysteine-labelling between 4 and 5 h p.i. The labelled viral proteins were identified according to their sizes. Viral protein 3C in the cell lysates was detected by western blotting.

We had demonstrated that EV71-infected cells generated a substantial amount of vsRNA1 through Dicer cleavage (Figures 1 and 2). We next examined the effect of vsRNA1 on EV71 infection. To determine whether EV71 infection generates functional vsRNA1, we blocked vsRNA1 in infected cells using a strategy similar to the miRNA sponge (50). As shown by Ebert *et al.* (50), this kind of sponge RNA competes with the RNA that binds to small RNA, ultimately blocking its function. We generated an anti-vsRNA1 sponge RNA containing 10 repeats of sequences that were the reverse complement of vsRNA1. We also designed an RNA containing 10 repeats of scrambled sequence as a control. The anti-vsRNA1 and control sponge RNA were transfected into EV71-infected cells. The viral yield in the anti-vsRNA1 sponge RNA-transfected cells was significantly higher than in the control RNA-transfected cells (Figure 4A). To investigate whether vsRNA1 influence viral infection at viral replication stage in cytoplasm, we co-transfected the anti-vsRNA1 sponge RNA or control RNA with an EV71 replicon encoding a firefly luciferase gene, which was used to quantify the viral protein expression. The luciferase signal from the replicon in the anti-vsRNA1-transfected cells was higher than in the control RNA-transfected cells at 9, 12 and 15 h after replicon transfection (Figure 4B), similar to the result in infected cells (Figure 4A). We also monitored newly synthesised proteins in anti-vsRNA1 sponge RNA-transfected cells by performing a ^35^S-methionine/cysteine labelling assay. The level of the labelled viral proteins in the anti-vsRNA1 sponge-transfected cells was higher than in the control RNA-transfected cells (Figure 4C, lane 4 compared with lane 3). Western blotting for a viral protein (3C^pro^) also confirmed the enhancement of viral protein expression by the anti-vsRNA1 sponge. The anti-vsRNA1 sponge did not affect host translation in mock-infected cells, which highlighted the specificity of the anti-vsRNA1 sponge for viral sequences (Figure 4C, lane 2 compared with lane 1). These results (Figure 4A–C) indicate that viral infection can generate functional vsRNA1, which downregulates viral yields and viral protein synthesis.

**Figure 5.**
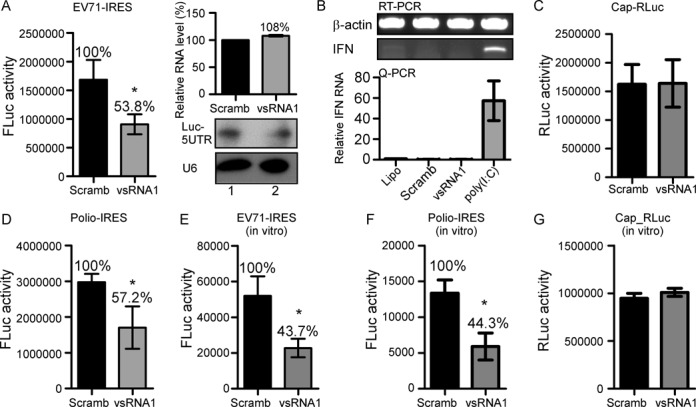
vsRNA1 inhibits viral IRES activity in vitro and in vivo. (**A**) Effect of vsRNA1 on EV71 IRES activity. SF268 cells were cotransfected with either the vsRNA1 mimic or scrambled RNA (Scramb) combined with a reporter RNA containing the EV71 IRES and the firefly luciferase gene (EV71 IRES-FLuc). The EV71 IRES-driven luciferase expression (FLuc activity) is represented by bars. Error bars represent standard deviations (*n* = 3, *P < 0.05, Student's *t*-test). The levels of the transfected reporter RNA (Luc-5′UTR) in the cells cotransfected with scrambled RNA (Scramb) or vsRNA1 were assessed using quantitative real-time RT-PCR (qPCR) (upper right panel) and northern blotting (lower right panel). (**B**) IFN-β mRNAs in Lipofectamine 2000-treated (Lipo) SF268 cells or cells transfected with scrambled RNA (Scramb); vsRNA1 and poly (I:C) were detected using qPCR. Error bars represent standard deviations (*n* = 3). (**C**) The effect of the vsRNA1 mimic on cap-dependent translation using a reporter RNA containing cap and Renilla luciferase (Cap-RLuc). (**D**) RNAs containing the poliovirus IRES and the firefly luciferase gene (polio IRES-FLuc) were also examined (*n* = 3, *P < 0.05, Student's *t*-test). The effects of vsRNA1 on EV71 IRES activity (**E**), poliovirus IRES activity (**F**), and cap-dependent translation (**G**) were also examined in vitro. Reporter RNAs incubated with the vsRNA1 mimic or scrambled RNA (Scramb) were examined in an in vitro translation assay using a mixture of SF268 cell lysate and rabbit reticulocyte lysate (RRL). The IRES- or cap-driven translation activity was determined according to the detected luciferase expression (FLuc activity and RLuc activity). Error bars represent standard deviations for three independent experiments (*P < 0.05, Student's *t*-test).

To further examine the effect of vsRNA1 on EV71 infection, we measured the viral titers in cells challenged with EV71, followed by transfection with a mimic vsRNA1 (the mimic RNA used in Figure 1E) or scrambled RNA. To simulate the vsRNA1, which is only produced during viral infection, and to avoid possible side effects resulting from long-term vsRNA1 expression, we transfected vsRNA1 or scrambled RNA into the cells at 1 h p.i. There were significantly lower viral yields in the cells transfected with vsRNA1 than in those with scrambled RNA (Figure 4D). We also cotransfected the mimic vsRNA1 or scrambled RNA with the EV71 replicon into cells. The luciferase signal was lower from the replicon in the vsRNA1-transfected cells than in the scrambled RNA-transfected cells (Figure 4E). The newly synthesised proteins in the vsRNA1-transfected cells were monitored using ^35^S-methionine/cysteine labelling. vsRNA1 inhibited viral protein synthesis (Figure 4F, lanes 3 and 4) but not host protein translation (mock, lanes 1–2). The effect of vsRNA1 was also confirmed by detecting 3C viral protein accumulation at 5 h p.i. using western blotting (Figure 5F, lanes 3–4, lower panel). Taken together, these results suggest that vsRNA1 plays a role in downregulating viral protein synthesis.

### vsRNA1 inhibits viral IRES activity

We showed that vsRNA1 inhibits both cytoplasmic replication reactions and the virus productivity of infected cells. Because the assays in Figure 4B, C, E and F demonstrated that viral protein synthesis was downregulated by vsRNA1, we hypothesised that vsRNA1 inhibits viral replication by modulating viral translation. To determine whether vsRNA1 inhibits viral translation directly, we examined the effects of vsRNA1 on EV71 IRES activity using a reporter assay. We transfected SF268 cells with a single-stranded, non-replicatable mRNA containing the EV71 IRES and the coding sequence for firefly luciferase (FLuc, a reporter) along with vsRNA1 or scrambled RNA (Scramb). The luciferase activity, which corresponded to EV71 IRES activity, was then measured. vsRNA1 inhibited up to 54% of EV71 IRES activity in the transfected cells compared with the scrambled RNA (Figure 5A, left panel). To determine whether vsRNA1 reduces IRES activity by depleting the RNA levels, we measured the RNA levels in transfected cells using real-time RT-PCR and northern blotting. Similar RNA levels were observed in the vsRNA1-transfected cells and the scramb-transfected cells (Figure 5A, right panel), suggesting that vsRNA1 repressed viral translation without degenerating the viral RNA. Certain viral RNAs can activate type I interferon (51,52) and cause global translational repression in infected cells (53,54). To determine whether vsRNA1 induces type I interferon, we examined interferon expression in the scramb-, vsRNA1-, and Polyinosinic-polycytidylic acid [poly (I:C)]-transfected cells. Transfecting poly (I:C), but not vsRNA1 or control RNA, induced type I interferon expression (Figure 5B). To determine whether vsRNA1 inhibits host cap-dependent translation, we transfected vsRNA1 and an in vitro-synthesised mRNA containing a 5′ cap structure and the coding sequence for Renilla luciferase into SF268 cells. vsRNA1 did not affect cap-dependent translation (Figure 5C), which was similar to the result that vsRNA1 did not affect cellular translation (Figure 4F). vsRNA1 also inhibited the IRES activity of poliovirus, another enterovirus (Figure 5D). Although we demonstrated that vsRNA1 did not affect cellular translation (Figures 4F and 5C), we were still concerned that vsRNA1 transfection might be toxic to the cells. To rule out possible bias produced by vsRNA1 transfection, we measured in vitro IRES activity to confirm the inhibitory effect of vsRNA1 on viral translation. In this assay, vsRNA1 or scrambled RNA was added to a mixture containing 20% RRL, a HeLa cell translation extract, and EV71–5′UTR-FLuc mRNA. Similar to the results obtained for transfected cells, vsRNA1 reduced EV71 IRES and polio-IRES activity to 44% of the activity in control RNA-transfected cells (Figure 5E and F). The same as the transfected cells, vsRNA1 did not affect cap-dependent translation in this in vitro assay (Figure 5G). Therefore, vsRNA1 specifically inhibited viral IRES activity in both live cells and in vitro reactions.

### vsRNA1 inhibits viral translation by targeting the IRES

**Figure 6.**
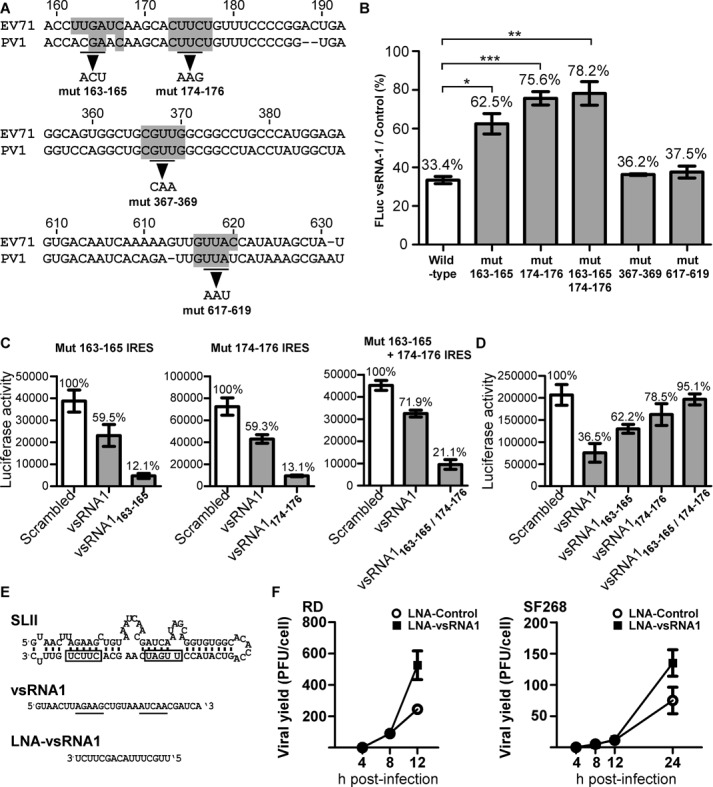
vsRNA1 target sites on IRES. (**A**) Predicted vsRNA1 target sites in the EV71 IRES (marked in grey) were selected based on conservation with poliovirus (PV1). Based on the prediction, we generated mutations in nt 163–165, 174–176, 367–369 and 617–619 of the EV71 IRES in the reporter RNA. (**B**) The inhibitory effects of vsRNA1 on WT and mutant IRES reporter RNAs. Each reporter RNA (including WT, mut 163–165, mut 174–176, mut 163–165, 174–175, mut 367–369 and mut 617–619) was treated with vsRNA1 or scrambled (control) RNA in an in vitro translation assay. The luciferase activity in the vsRNA1 treatment assay was compared to the activity in the scrambled RNA treatment assay, and the percentage of IRES activity under vsRNA1 treatment was calculated separately. Error bars represent standard deviations for three independent experiments (*P < 0.05, **P < 0.01, ***P < 0.001, Student's *t*-test). (**C**) Mutant IRES reporter RNAs were treated with the scrambled or vsRNA1 (or modified vsRNA1) that corresponded to each target site mutant (vsRNA1_163_–_165_ to mut 163–165 IRES, vsRNA1 174–176 to mut 174–176 RNA and vsRNA1 163–165/174–176 to mut 163–165 + 174–176) in the in vitro translation assays. (**D**) WT IRES reporter RNA was treated with scrambled RNA or vsRNA1 (or modified vsRNA1) in an in vitro translation assay. Error bars represent standard deviations for three independent experiments. (**E**) LNA against vsRNA1 was generated complementary to the active sites (underlined) on vsRNA1 that target stem-loop II (SLII) of the EV71 5′UTR (the boxes indicate the vsRNA1 target sites). Effect of anti-vsRNA1 LNA on EV71 viral growth. (**F**) EV71-infected RD and SF268 cells (MOI of 5) were transfected with LNA-vsRNA1 or LNA-control. After viral adsorption, viruses from the debris and the supernatant were collected at the indicated times p.i. The viral yields were determined using plaque assays. Error bars represent standard deviations for duplicated assays.

vsRNA1 inhibited viral IRES activity (Figure 5). To investigate whether vsRNA1 can inhibit IRES activity by targeting a specific RNA region, we searched for potential vsRNA1 target sites using the entire IRES sequence. We identified a total of 10 potential target sites in the EV71 IRES. Such sites contained five contiguous nucleotides that were the reverse complement of the vsRNA1 sequence. Because vsRNA1 also inhibited polio IRES activity (Figure 5D and F), we expected the sequences of the vsRNA1 target sites on the polio IRES to be similar to those on the EV71 IRES. We compared the sequences of EV71 and polio IRES to narrow down the potential target sites. Four of the 10 potential target sites, located at nt 162–166, 173–177, 366–370 and 616–620 of the EV71 5′UTR, were similar to the sequences of the polio IRES at the same locations (Figure 6A, grey boxes). We selected these four potential target sites for further study. To determine whether the four target sites in the IRES are crucial for the inhibitory effect of vsRNA1, we mutated the three central nucleotides of each potential target site in the EV71 5′UTR-FLuc RNA (Figure 6A). All of the mutated EV71 5′UTR Fluc RNAs were checked for their IRES activity using the wild-type group as the reference category (Supplementary Figure S6). An in vitro translation assay was performed to test the inhibitory effects of vsRNA1 on the activities of the mutated reporter RNAs. Differently from the WT IRES that was inhibited by vsRNA1, EV71 IRESs containing mutations in nt 163–165 (mut 163–165), nt 174–176 (mut 174–176) and double mutations (mut 163–165 and 174–176) were found to be resistant to the inhibitory effect of vsRNA1 (Figure 6B). Mutations in nt 367–369 (mut 367–369) and nt 617–619 (mut 617–619) of the IRES had no effect on vsRNA1 resistance. To further confirm that mut 163–165, mut 174–176 and mut 163–165 + 174–176 became resistant to vsRNA1 by losing the target sites in the IRES, we generated vsRNA1s with sequence modifications corresponding to the mutated IRES sites (vsRNA1_163_–_165_, vsRNA1_174_–_176_ and vsRNA1_163_–_165/174–176_). Each modified vsRNA1 substantially inhibited the corresponding mutant IRES (80–90% inhibition) (Figure 6C). We also tested the inhibitory effects of the modified vsRNA1s on WT IRES activity. In vsRNA1_163_–_165_ and vsRNA1_174_–_176_, the ability to inhibit the WT IRES was partially lost (20%–30% inhibition) (Figure 6D). vsRNA1_163_–_165/174–176_ did not inhibit the WT IRES, suggesting that nt 162–166 and nt 173–177 are the target sites for the inhibition of IRES activity by vsRNA1. From these results, we concluded that vsRNA1 inhibits IRES activity by targeting specific sequences on stem-loop II of the IRES (Figure 6E, upper panel). Notably, more than 80% of the vsRNA1 deep sequencing reads contained both of these active sequences (Supplementary Figure S7). To verify that the target sites identified in the in vitro assay were valid in infected cells, we designed a tiny LNA (anti-vsRNA1 LNA) to disrupt the association between the target sites and vsRNA1 (Figure 6E, lower panel). Based on the results obtained by Obad *et al.*, tiny LNA was able to solely target the seed region of the miRNA, without directly affecting the miRNA-targeted mRNA (55). We also confirmed that our anti-vsRNA1 LNA did not target the EV71 IRES directly in an IRES activity assay (Supplementary Figure S8). We transfected the anti-vsRNA1 LNA or scrambled LNA into EV71-infected cells. The results demonstrated that the anti-vsRNA1 LNA enhanced viral infection in both RD and SF268 cells (Figure 6F), providing further confirmation of the vsRNA1 target sites identified on the EV71 IRES in vivo.

## DISCUSSION

**Figure 7.**
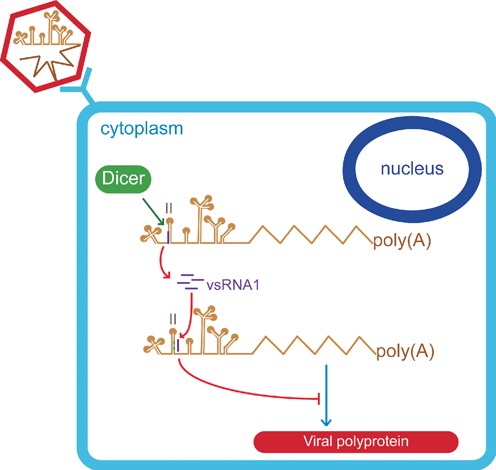
Proposed model for the generation and function of vsRNA1. During EV71 infection, a small proportion of viral RNA is processed by Dicer, and vsRNA1 is generated from the IRES (purple). vsRNA1 targets stem-loop II of the viral IRES (marked in green) and downregulates the translation of viral proteins.

This study showed that EV71 uses Dicer to generate small RNAs in mammalian cells. One of these vsRNAs, vsRNA1, downregulated IRES activity by targeting viral RNA (summarised in Figure 7). It has been debated whether a cytoplasmic RNA virus in mammals can generate functional small RNAs (38); however, our results show that this type of virus can generate substantial amounts of vsRNA that exhibits notable functions.

Because positive-stranded RNA viruses use the same RNA as a template for both translation and replication, strategies that control the switch between viral translation and genome replication are crucial (24,25). For example, poliovirus uses a host protein, Poly(rC)-binding protein 2 (PCBP2), to regulate both viral translation and replication (56–61). Viral proteases cleave host proteins, which can enhance viral translation (62–64). This study revealed a potential novel mechanism by which positive-sense RNA viruses control their translation: generating vsRNAs and self-regulating IRES activity. However, we cannot rule out the possibility that the host may utilize such vsRNAs as a defence mechanism against EV71 infections. Therefore, the role of vsRNAs in virus infection deserves further scrutiny.

Recently, non-canonical cytoplasmic mechanisms have been reported to contribute to small RNA biogenesis (11–12,65). Dicer might be involved in these processes (14), which correlates with our findings that Dicer is associated with the generation of vsRNAs (Figures 2 and 3). The substrate of Dicer is generally a stem-loop structure with both 5′ and 3′ ends (66). However, the EV71 5′UTR does not contain such a structure, which is instead characterised by long flanking regions at both ends of the stem-loop. Because non-canonical Dicer substrates have been previously identified (67–69), the mechanisms by which this enzyme can cleave the EV71 5′UTR RNA into vsRNA1 seem worthy of investigation. We detected vsRNA1 as an RNA fragment of 29 nt (Figure 1E), which is longer than the small RNA generated by canonical Dicer processing (22 nt). However, the bulged nucleotides on the stem-loops of pre-mRNAs can increase the length of miRNA that Dicer is able to process (70,71). The vsRNA1 generation site contains a bulged structure (Figure 1C), which might explain how Dicer cleavage generates a long small RNA.

Parameswaran *et al.* detected low-abundance vsRNA reads in infected cells using deep sequencing (14). Coincidently, we also detected limited numbers of vsRNA1 reads in infected cells using deep sequencing (Figure 1A). To our surprise, a northern blot, which was performed to confirm the absolute level of vsRNA1, showed that at least 10^5^ vsRNA1 copies were generated from a single infected cell (Figure 1E). Such inconsistent results between deep sequencing and other RNA detection methods have also been found in previous studies (72,73). Factors such as different PCR protocols, ligation steps in deep sequencing, or the modification of the small RNA terminus may bias the quantification of small RNAs (73). We assume that these types of bias caused our deep sequencing results to include underestimated levels of vsRNA. We cannot exclude the possibility that vsRNAs can undergo further unidentified modifications that should be the subject of future research. However, we demonstrated that infected cells could generate vsRNA1 in amounts sufficient for activity (Figure 4), which seems to suggest that infected cells contain a substantial level of vsRNA1. Besides vsRNA1, we also detected high amounts of vsRNAs derived from regions other than the 5′UTR of the viral genomic RNA (Supplementary Figure S9). Despite being outside the scope of the current report, the potential role played by these vsRNAs in virus infection is worthy of further scrutiny.

The function of small RNAs invariably requires the interaction with cellular proteins, including Argonaute 2 (Ago2) (39–41). The question as to whether any cellular protein is specifically required for vsRNA1 function is important for characterizing the biological role of this molecule. Although our data indicated that vsRNA1 can be associated with Ago2 (Supplementary Figure S2), the experiments conducted after Ago2 depletion did not support the hypothesis that this molecule is involved in either vsRNA1 biogenesis (Supplementary Figure S4) or in the inhibitory effect on IRES activity (Supplementary Figure S10). Therefore, the potential role of both vRNA1-associated Ago2 and/or other vsRNA1-associated proteins warrants further investigation.

Besides the identification of vsRNA1-associated proteins, it seems relevant to investigate whether vsRNA1 may affect the binding of ITAFs to the EV71 IRES, especially at the regions targeted by vsRNA1 (stem-loop II of the IRES). Among the ITAFs that can bind to the SLII of EV71 IRES (KSRP, hnRNPA1, PTB) (26–27,74), our preliminary results indicated that vsRNA1 can both enhance KSRP binding to the IRES (Supplementary Figure S11) and increase the amount of KSRP sedimented in the fractions containing 40S and 60S ribosomal subunits, as compared with a scrambled RNA sequence in a ribosome assembly experiment (Supplementary Figure S12). KSRP has been shown to act as a negative regulator of EV71 IRES activity (26,75). The current observations suggest that vsRNA1 may inhibit IRES activity by recruiting a negative regulator to the translational complex. The exact roles played by KSRP in vsRNA1 function and the detailed mechanisms of KSRP recruitment elicited by vsRNA1 deserve further scrutiny.

Small RNAs are likely to play a role in determining the tissue tropism of a given virus. For example, a host miRNA122 targets the 5′ noncoding region of the hepatitis C virus (HCV) and enhances viral replication in hepatocytes (76,77). Our deep-sequencing results demonstrated significant differences (both in terms of levels and species) in vsRNAs expression between RD (rhabdomysosarcoma) and SF268 (glioblastoma) cells (Supplementary Figure S1), suggesting a potential mechanism for viral tissue tropism. Because blocking host miRNA122 using LNA represents a novel treatment strategy for HCV infection (78,79), our results may help developing novel potential antiviral strategies in the prevention of EV71-associated neuropathogenesis.

In summary, our study demonstrates the ability of a cytoplasmic RNA virus to generate functional vsRNA in mammalian cells. In addition, we demonstrate a novel mechanism for the self-regulation of positive-stranded RNA viral translation, which includes the generation of a vsRNA that targets the IRES. Importantly, our data disclose a previously unknown role of vsRNA.

## SUPPLEMENTARY DATA

Supplementary Data are available at NAR Online.

SUPPLEMENTARY DATA
