# Supplementary material for: A cytoplasmic RNA virus generates functional viral small RNAs and regulates viral IRES activity in mammalian cells
Source: Nucleic Acids Res. 2014 Oct 28;42(20):12789–805. doi: 10.1093/nar/gku952 (PMC4227785; doi:10.1093/nar/gku952)
Supplement: SUPPLEMENTARY DATA [file supp_42_20_12789__index.html]

A cytoplasmic RNA virus generates functional viral small RNAs and regulates viral IRES activity in mammalian cells — A cytoplasmic RNA virus generates functional viral small RNAs and regulates viral IRES activity in mammalian cells — SUPPLEMENTARY DATA 

# A cytoplasmic RNA virus generates functional viral small RNAs and regulates viral IRES activity in mammalian cells

## SUPPLEMENTARY DATA

**Files in this Data Supplement:**

- SUPPLEMENTARY DATA
