# Supplementary material for: A cytoplasmic RNA virus generates functional viral small RNAs and regulates viral IRES activity in mammalian cells
Source: Nucleic Acids Res. 2014 Oct 28;42(20):12789–805. doi: 10.1093/nar/gku952 (PMC4227785; doi:10.1093/nar/gku952)
Supplement: SUPPLEMENTARY DATA [file supp_gku952_nar-01868-v-2014-File009.pdf]

Supplementary Figures

Figure S1

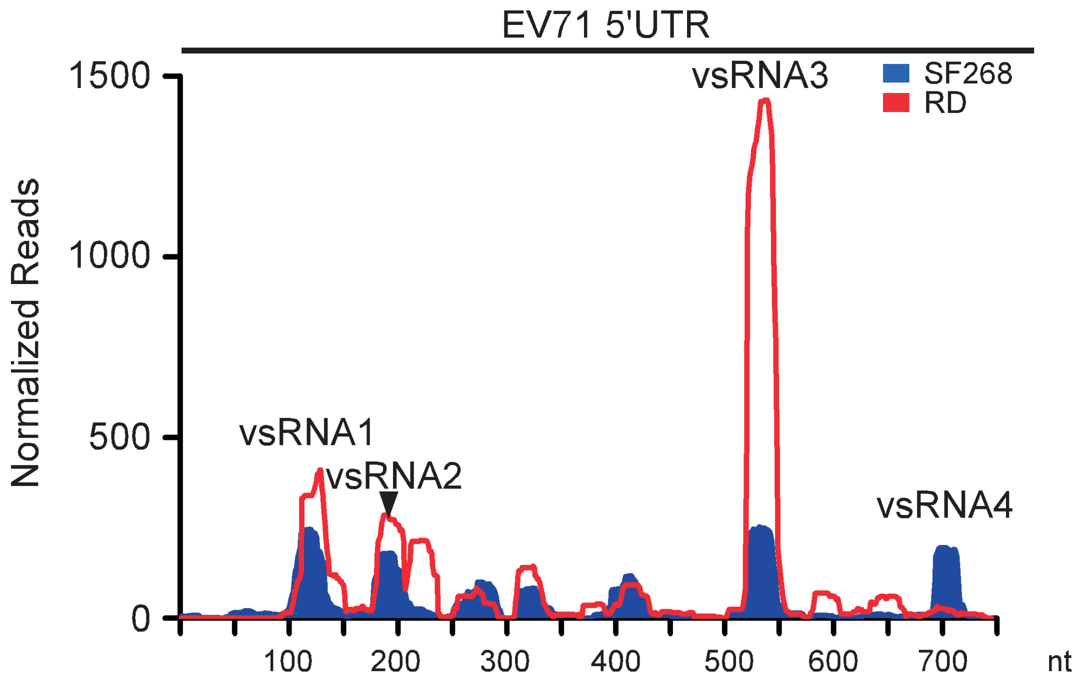

Figure S1. Virus-derived small RNAs (vsRNAs) in SF268 (blue) and RD (red) cells infected with EV71. The vsRNAs in the infected cells were sequenced using Illumina technology. The position distributions and abundance (normalised reads) of that sequenced vsRNAs that perfectly matched the EV71 5'UTR are shown. The vsRNA1, vsRNA2, vsRNA3 and vsRNA4, which were detected in SF268-infected cells, were indicated.

Figure S2

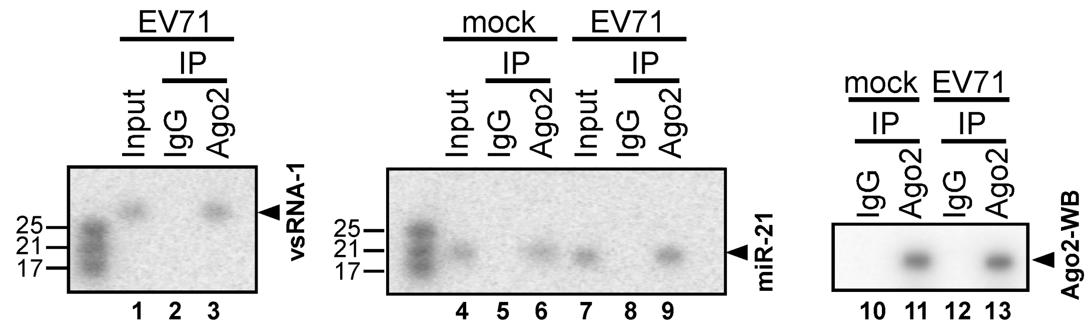

Figure S2. Ago2 associates with vsRNA1. Ago2 was immunoprecipitated, and the associated vsRNA1 and miR-21 in mock- and EV71-infected cells were detected by northern blotting. RNA pulled down by IgG alone was the control. Ago2 protein pulled down in mock and infected cells was detected by western blotting (Ago2-WB).

Figure S3

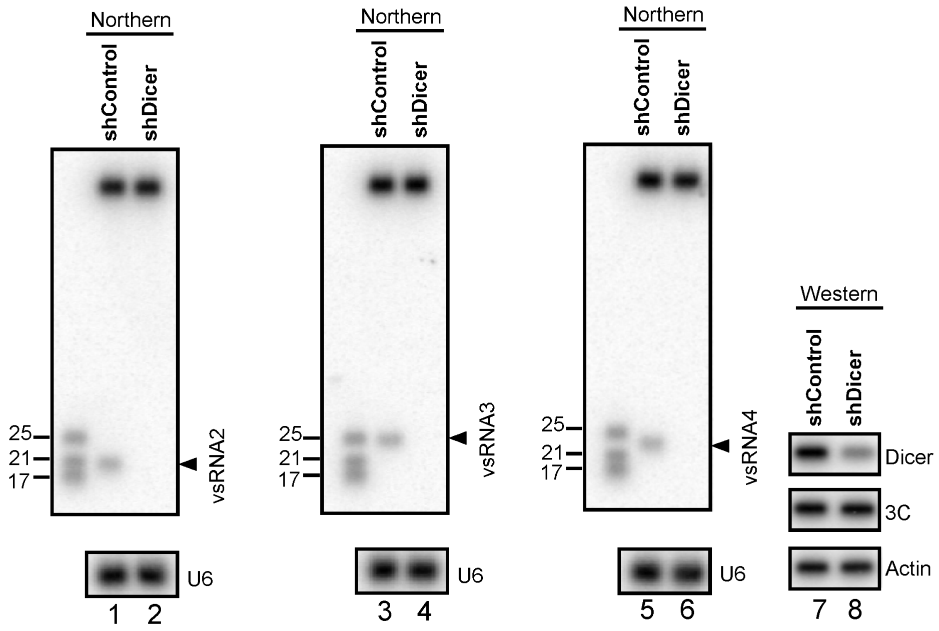

Figure S3. vsRNA2, vsRNA3 and vsRNA4 in Dicer-depleted cells. vsRNA2, vsRNA3, and vsRNA4 in SF268 cells transfected with the control plasmid (shControl) or a plasmid expressing shRNA against Dicer (shDicer) were detected using specific probes. The Dicer, viral 3C protein levels in these cells were detected by western blotting.

Figure S4

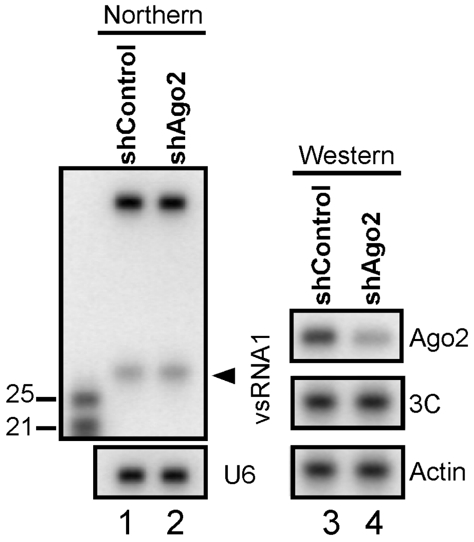

Figure S4. vsRNA1 in Ago2-depleted cells infected with EV71. vsRNA1 in SF268 cells transfected with the control plasmid (shControl) or a plasmid expressing shRNA against Argonaute 2 (shAgo2) was detected using a vsRNA1 probe. The Argonaute 2, viral 3C protein levels in these cells were detected by western blotting.

Figure S5

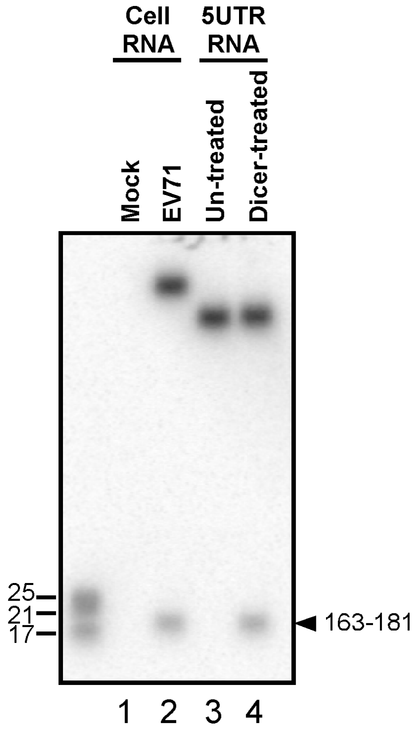

Figure S5. Small RNA fragment 163-181 in EV71-infected cells and in Dicer-treated 5'UTR RNA reaction. A probe against 163-181 nt of the EV71 5'UTR was used to detect the 163-181 RNA fragment generated by EV71-infected cells (Cell RNA) and by synthetic EV71 5'UTR RNA after Dicer treatment (Dicer-treated). RNA isolated from mock-infected cells and from a synthetic 5'UTR without Dicer treatment (Un-treated) served as negative controls.

Figure S6

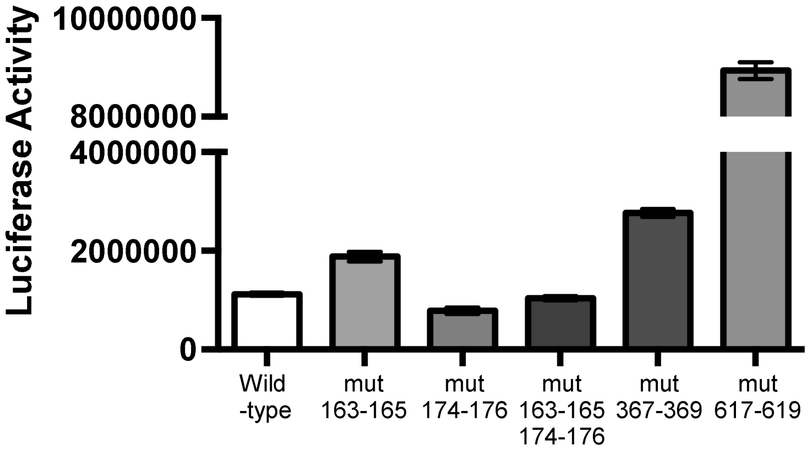

Figure S6. Luciferase activities driven by wild-type, mut 163-165, mut 174-176, mut 163-165 + 174-176, mut 367-369, mut 617-619 IRESs. The luciferase activity of each reporter RNA containing either wild-type or mutant IRES (mut 163-165, mut 174-176, mut 163-165 + 174-176, mut 367-369, and mut 617-619) was examined using an *in vitro* translation assay. The average of three independent experiments is shown; error bars indicate standard deviations.

Figure S7

```
    TTGATC-A-A--GC-A-CTTCT
1   ‹TTGATT-T-A---C-AGCTTCT›
    ‹TTGATT-T-A---C-AGCTTCT›
    ‹TTGATT-T-A---C-AGCTTCT›
    ‹TTGATT-T-A---C-AGCTTCT›
    ‹TTGATTTACA--GC
    ‹TTGATT-T-A---C-AGCTTC
    ‹TTGATT-T-A---C-AGCTTCT›
    ‹TTGATT-T-A---C-AGCTTCT›
    ‹TTGATT-T-A---C-AGCTTCT›
    ‹TTGATT-T-A---C-AGCTTCT›
11  ‹TTGATT-T-A---C-AGCTTCT›
    ‹TTGATT-T-A---C-AGCTTCT›
    ‹TTGATT-T-A---C-AGCTTCT›
    ‹TTGATT-T-A---C-AGCTTCT
    ‹TTGATT-T-A---C-AGCTTCT›
    ‹TTGATT-T-A---C-AGCTTCT›
    ‹TTGATT-T-A---C-AGCTTCT›
    ‹TTGATT-T-A---C-AGCTTCT›
    ‹TTGATT-T-A---C-AGCTTCT›
    ‹TTGATT-T-A---C-AGCTTCT›
21  ‹TTGATT-T-A---C-AGCTTCT›
    ‹TTGATT-T-A---C-AGCTTCT›
    ‹TTGATT-T-A---C-AGCTTCT›
    ‹TTGATT-T-A---C-AGCTTCT›
    ‹TTGATT-T-A---C-AGCTTCT›
    ‹TTGATT-T-A---C-AGCTTCT›
    ‹TTGATT-T-A---C-AGCTTCT›
    ‹TTGATT-T-A---C-AGCTTCT›
    ‹TTGATT-T-A---C-AGCTTCT›
    ‹TTGATT-T-A---C-AGCTTCT›
31  ‹TTGATT-T-A---C-AGCTTCT›
    ‹TTGATT-T-A---C-AGCTTCT›
    ‹TTGATT-T-A---C-AGCTTCT›
    ‹TTGATT-T-A---C-AGCTTCT›
    ‹TTGATT-T-A---C-AGCTTCT›
    ‹TTGATT-T-A---C-AGCTTCT›
    ‹TTGATT-T-A---C-AGCTTCT›

    TTGATC-A-A--GC-A-CTTCT
    ‹TTGATT-T-A---C-AGCTTCT›
    ‹TTGATT-T-A---C-AGCTTCT›
    ‹TTGATT-T-A---C-AGCTTCT›
41  ‹TTGATT-T-A---C-AGCTTCT›
    ‹TTGATT-T-A---C-AGCTTCT›
    ‹TTGATT-T-A---C-AGCTTCT›
    ‹TTGATT-T-A---C-AGCTTCT›
    ‹TTGATT-T-A---C-AGCTTCT›
    ‹TTGATT-T-A---C-AGCTTCT›
    ‹TTGATT-T-A---C-AGCTTCT›
    ‹TTGATT-T-A---C-AGCTTCT›
    ‹TTGATT-T-A---C-AGCTTCT›
    ‹TTGATT-T-A---C-AGCTTCT›
    ‹TTGATT-T-A---C-AGCTTCT›
51  ‹TTGATT-T-A---C-AGCTTCT›
    ‹TTGATT-T-A---C-AGCTTCT›
    ‹TTGATT-T-A---C-AGCTTCT›
    ‹TTGATT-T-A---C-AGCTTCT›
    ‹TTGATT-T-A---C-AGCTTCT›
    ‹TTGATT-T-A---C-AGCTTCT›
    ‹TTGATT-T-A---C-AGCTTCT›
    ‹TTGATT-T-A---C-AGCTTCT›
    ‹TTGATT-T-A---C-AGCTTCT›
    ‹TTGATT-T-A---C-AGCTTCT›
61  ‹TTGATT-T-A---C-AGCTTCT›
    ‹TTGATT-T-A---C-AGCTTCT›
    ‹TTGATT-T-A---C-AGCTTCT›
    ‹TTGATT-T-A---C-AGCTTCT›
    ‹TTGATT-T-A---C-AGCTTCT›
    ‹TTGATT-T-A---C-AGCTTCT›
    ‹TTGATT-T-A---C-AGCTTCT›
    ‹TTGATT-T-A---C-AGCTTCT›
    ‹TTGATT-T-A---C-AGCTTCT›
    ‹TTGATT-T-A---C-AGCTTCT›
71  ‹TTGATT-T-A---C-AGCTTCT›
    ‹TTGATT-T-A---C-AGCTTCT›
    ‹TTGATT-T-A---C-AGCTTCT›
    ‹TTGATT-T-A---C-AGCTTCT›

    TTGATC-A-A--GC-A-CTTCT
    ‹TTGATT-T-A---C-AGCTTCT›
    ‹TTGATT-T-A---C-AGCTTCT›
    ‹TTGATT-T-A---C-AGCTTCT›
    ‹TTGATT-T-A---C-AGCTTCT›
    ‹TTGATT-T-A---C-AGCTTCT›
    ‹TTGATT-T-A---C-AGCTTCT›
81  ‹TTGATT-T-A---C-AGCTTCT›
    ‹TTGATT-T-A---C-AGCTTCT›
    ‹TTGATT-T-A---C-AGCTTCT›
    ‹TTGATT-T-A---C-AGCTTCT›
    ‹TTGATT-T-A---C-AGCTTCT›
    ‹TTGATT-T-A---C-AGCTTCT›
    ‹TTGATT-T-A---C-AGCTTCT›
    ‹TTGATT-T-A---C-AGCTTCT›
    ‹TTGATT-T-A---C-AGCTTCT›
    ‹TTGATT-T-A---C-AGCTTCT›
91  ‹TTGATT-T-A---C-AGCTTCT›
    ‹TTGATT-T-A---C-AGCTTCT›
    ‹TTGATT-T-A---C-AGCTTCT›
    ‹TTGATT-T-A---C-AGCTTCT›
    ‹TTGATT-T-A---C-AGCTTCT›
    ‹TTGATT-T-A---C-AGCTTCT›
    ‹TTGATT-T-A---C-AGCTTCT›
    ‹TTGATT-T-A---C-AGCTTCT›
    ‹TTGATT-T-A---C-AGCTTCT›
    ‹TTGATT-T-A---C-AGCTTCT›
101 ‹TTGATT-T-A---C-AGCTTCT›
    ‹TTGATT-T-A---C-AGCTTCT›
    ‹TTGATT-T-A---C-AGCTTCT›
    ‹TTGATT-T-A---C-AGCTTCT›
    ‹TTGATT-T-A---C-AGCTTCT›
    ‹TTGATT-T-A---C-AGCTTCT›
    ‹TTGATT-T-A---C-AGCTTCT›
    ‹TTGATT-T-A---C-AGCTTCT›
    ‹TTGATT-T-A---C-AGCTTCT›
    ‹TTGATT-T-A---C-AGCTTCT›
111 ‹TTGATT-T-A---C-AGCTTCT›
```

```
TTGATC-A-A--GC-A-CTTCT
‹TTGATT-T-A---C-AGCTTCT›
‹TTGATT-T-A---C-AGCTTCT›
‹TTGATT-T-A---C-AGCTTCT›
‹TTGATT-T-A---C-AGCTTCT›
‹TTGATT-T-A---C-AGCTTCT›
‹TTGATT-T-A---C-AGCTTCT›
‹TTGATT-T-A---C-AGCTTCT›
‹TTGATT-T-A---C-AGCTTC
‹TTGATT-T-A---C-AGCTTC
121 ‹TTGATT-T-A---C-AGCTTC
‹TTGATT-T-A---C-AGCTTC
‹TTGATT-T-A---C-AGCTT
‹TTGATT-T-A---C-AGCTTCT›
‹TTGATT-T-A---C-AGCTTCT›
‹TTGATT-T-A---C-AGCTTCT›
‹TTGATT-T-A---C-AGCTTCT›
‹TTGATT-T-A---C-AGCTTCT›
‹TTGATT-T-A---C-AGCTTCT›
‹TTGATT-T-A---C-AGCTTCT›
131 ‹TTGATT-T-A---C-AGCTTCT›
‹TTGATT-T-A---C-AGCTTCT›
‹TTGATT-T-A---C-AGCTTCT›
‹TTGATT-T-A---C-AGCTTCT›
‹TTGATT-T-A---C-AGCTTCT›
‹TTGATT-T-A---C-AGCTTCT›
‹TTGATT-T-A---C-AGCTTCT›
‹TTGATT-T-A---C-AGCTTCT›
‹TTGATT-T-A---C-AGCTTCT›
‹TT-A-C-A----GC----TTCT›
141 ‹TT-A-C-A----GC----TTCT›
‹TT-A-C-A----GC----TTCT›
‹TT-A-C-A----GC----TTCT›
‹TT-A-C-A----GC----TTCT›
‹TT-A-C-A----GC----TTCT›
‹TTGATT-T-A---C-AGCTTCT›
‹TTGATT-T-A---C-AGCTTCT›
‹TT-A-C-A----GC----TTCT›
```

```
TTGATC-A-A--GC-A-CTTCT
‹TTGATT-T-A---C-AGCTTCT›
‹TTGATT-T-A---C-AGCTTCT›
151 ‹TTGATT-T-A---C-AGCTTCT›
‹TTGATT-T-A---C-AGCTTCT›
‹TTGATT-T-A---C-AGCTTCT›
‹TTGATT-T-A---C-AGCTTCT›
‹TTGATT-T-A---C-AGCTTCT›
‹TTGATT-T-A---C-AGCTTCT›
‹TTGATT-T-A---C-AGCTTCT›
‹TTGATT-T-A---C-AGCTTCT›
‹TTGATT-T-A---C-AGCTTCT›
‹TTGATT-T-A---C-AGCTTCT›
161 ‹TTGATT-T-A---C-AGCTTCT›
‹TTGATT-T-A---C-AGCTTCT›
‹TTGATT-T-A---C-AGCTTCT›
‹TTGATT-T-A---C-AGCTTCT›
‹TTGATT-T-A---C-AGCTTCT›
‹TTGATT-T-A---C-AGCTTCT›
‹TT-A-C-A----GC----TTCT›
‹TTGATT-T-A---C-AGCTTCT›
‹TTGATT-T-A---C-AGCTTCT›
‹TTGATT-T-A---C-AGCTTCT›
171 TTGATT-T-A---C-AGCTTCT›
TTGATT-T-A---C-AGCTTCT›
TGATT-T-A---C-AGCTTCT›
TGATT-T-A---C-AGCTTCT›
TGATT-T-A---C-AGCTTCT›
TGATT-T-A---C-AGCTTCT›
TGATT-T-A---C-AGCTTCT›
TGATT-T-A---C-AGCTTCT›
TGATT-T-A---C-AGCTTCT›
TGATT-T-A---C-AGCTTCT›
181 TGATT-T-A---C-AGCTTCT›
TGATT-T-A---C-AGCTTCT›
TGATT-T-A---C-AGCTTCT›
GATT-T-A---C-AGCTTCT›
GATT-T-A---C-AGCTTCT›
```

```
TTGATC-A-A--GC-A-CTTCT
GATT-T-A---C-AGCTTCT›
GATT-T-A---C-AGCTTCT›
GATT-T-A---C-AGCTTCT›
GATT-T-A---C-AGCTTCT›
GATT-T-A---C-AGCTTCT›
191 GATT-T-A---C-AGCTTCT›
GATT-T-A---C-AGCTTCT›
GATT-T-A---C-AGCTTCT›
GATT-T-A---C-AGCTTCT›
GATT-T-A---C-AGCTTCT›
GATT-T-A---C-AGCTTCT›
GATT-T-A---C-AGCTTCT›
GATT-T-A---C-AGCTTCT›
GATT-T-A---C-AGCTTCT›
GATT-T-A---C-AGCTTCT›
201 GATT-T-A---C-AGCTTCT›
GATT-T-A---C-AGCTTCT›
GATT-T-A---C-AGCTTCT›
GATT-T-A---C-AGCTTCT›
‹AGCTTCTA-A--GTTA-CTTCG
‹AGCTTCTA-A--GTTA-CTTCG
‹AGCTTCTA-A--GTTA-CTTC
‹AGCTTCTA-A--GTTA-CTTCG›
‹AGCTTCTA-A--GTTA-CTTC
‹AGCTTCTA-A--GTTA-CTT
211 ‹AGCTTCTA-A--GTTA-CTT
‹AGCTTCTA-A--GTTA-CTTCG›
‹AGCTTCTA-A--GTTA-CTTCG›
‹AGCTTCTA-A--GTTA-CTTCG›
‹AGCTTCTA-A--GTTA-CTTCG›
‹AGCTTCTA-A--GTTA-CTTCG
‹AGCTTCTA-A--GTTA-CTTCG
AGCTTCTA-A--GTTA-CTTCG›
AGCTTCTA-A--GTTA-CTTCG›
AGCTTCTA-A--GTTA-CTTCG›
221 GCTTCTA-A--GTTA-CTTCG›
GCTTCTA-A--GTTA-CTTCG›
```

TTGATC-A-A--GC-A-CTTCT

GCTTCTA-A--GTTA-CTTCG

GCTTCTA-A--GTTA-CTTC

Figure S7 Sequences of vsRNA1 (reverse complement) which match to target sites on EV71 IRES. The sequences of vsRNA1 reads (reverse complement; red) from EV71-infected SF268 cells corresponding to the sequence of EV71 IRES which contains vsRNA1 target sites (box) were listed. Within the 243 reads of vsRNA1 (+ sense) identified by deep sequencing, 19 reads of vsRNA1 were unable to match to this IRES region, and were not listed here. This comparison shows that 222 of the total vsRNA1 reads (243, includes the 19 unmatched reads) contain sequences match to at least 1 target site (91.4%), and 203 reads contain sequences match to both of the 2 target sites (83.5%).

Figure S8

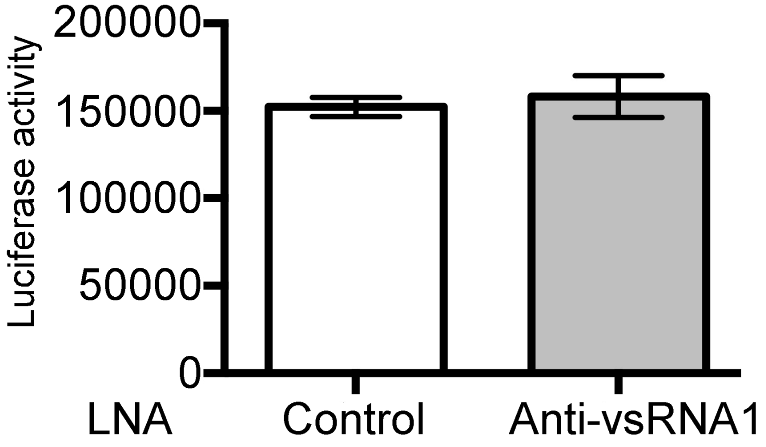

Figure S8. Effect of LNA-vsRNA1 on EV71 IRES activity. RD cells were cotransfected with either the LNA-control or anti-vsRNA1 LNA combined with the EV71 IRES reporter RNA. The EV71 IRES-driven luciferase expression is reported accordingly. The average of three independent experiments is shown; error bars indicate standard deviations.

Figure S9

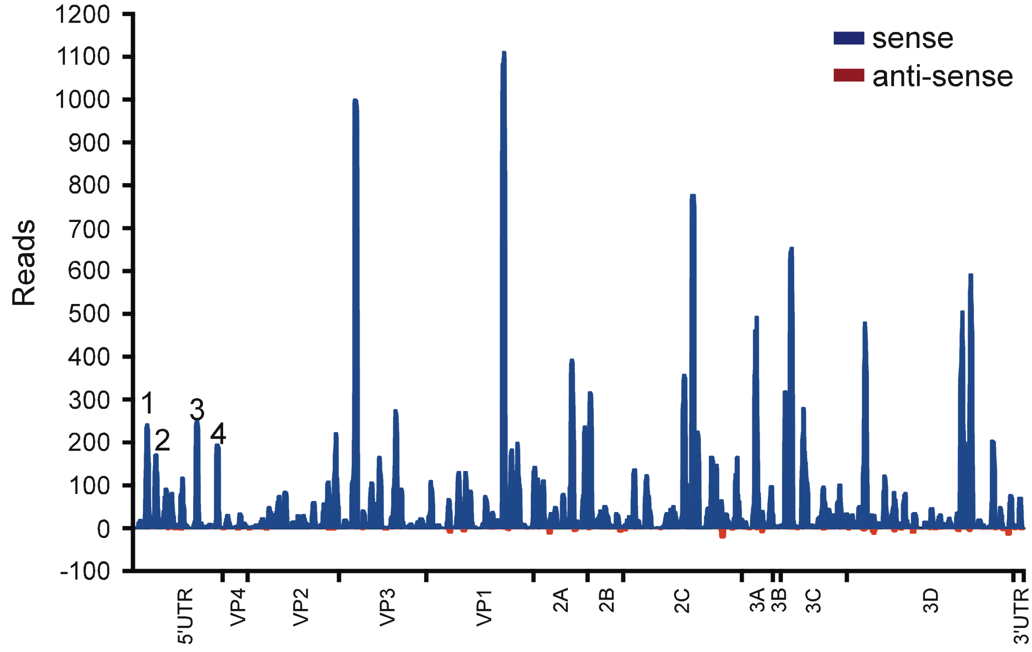

Figure S9. Virus-derived small RNAs (vsRNAs) in SF268 cells infected with EV71. The vsRNAs in infected cells were sequenced using Illumina technology. The position distributions and abundance (Reads) of that sequenced vsRNAs that perfectly matched the EV71 genome are shown. The vsRNA1, vsRNA2, vsRNA3 and vsRNA4 derived from EV71 5'UTR region were indicated.

Figure S10

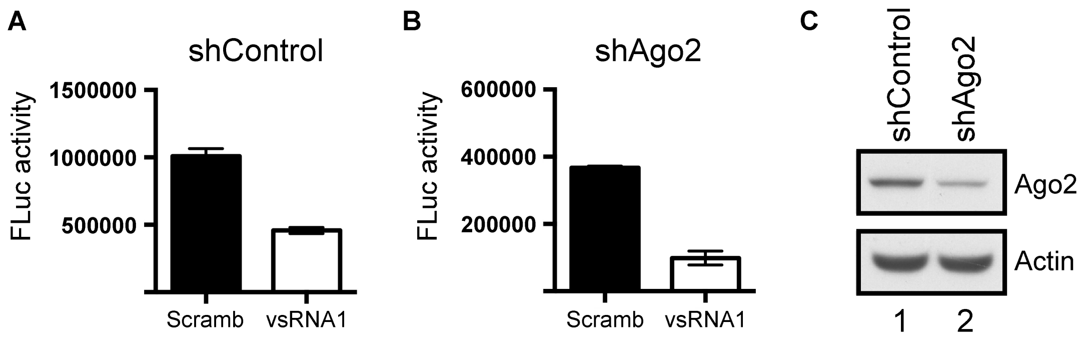

Figure S10. Effect of vsRNA1 on EV71 IRES activity in Ago2-depleted cell lysates. EV71 IRES Reporter RNAs incubated with vsRNA1 mimic or scrambled RNA (Scramb) were examined using an *in vitro* translation assay. To this aim, cell lysates harvested from Ago2-depleted cells (shAgo2) or control vector-transfected cells (shControl) were used. The average of three independent experiments is shown; error bars indicate standard deviations. The efficiency of Ago2 depletion was examined by Western blot.

Figure S11

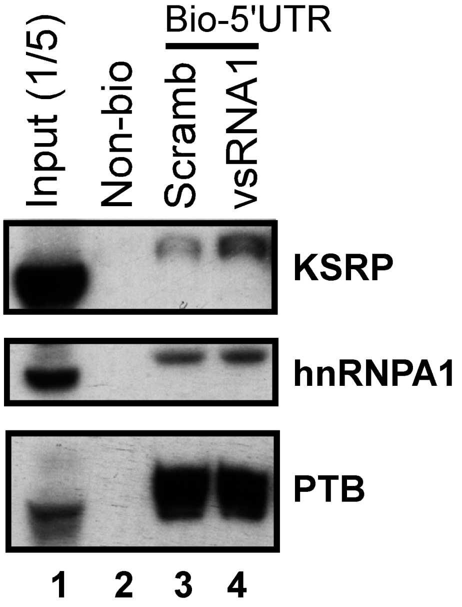

Figure S11. Effect of vsRNA1 on KSRP, hnRNPA1 and PTB binding to EV71 5'UTR RNA. Proteins from SF268 cell lysates were incubated with unlabeled (Non-bio) or biotin-labelled (Biotin) EV71 5'UTR RNA and with vsRNA1 or scrambled RNA (scramb). The 5'UTR-bound proteins from lysates were pulled down using streptavidin beads and analyzed by Western blotting.

Figure S12

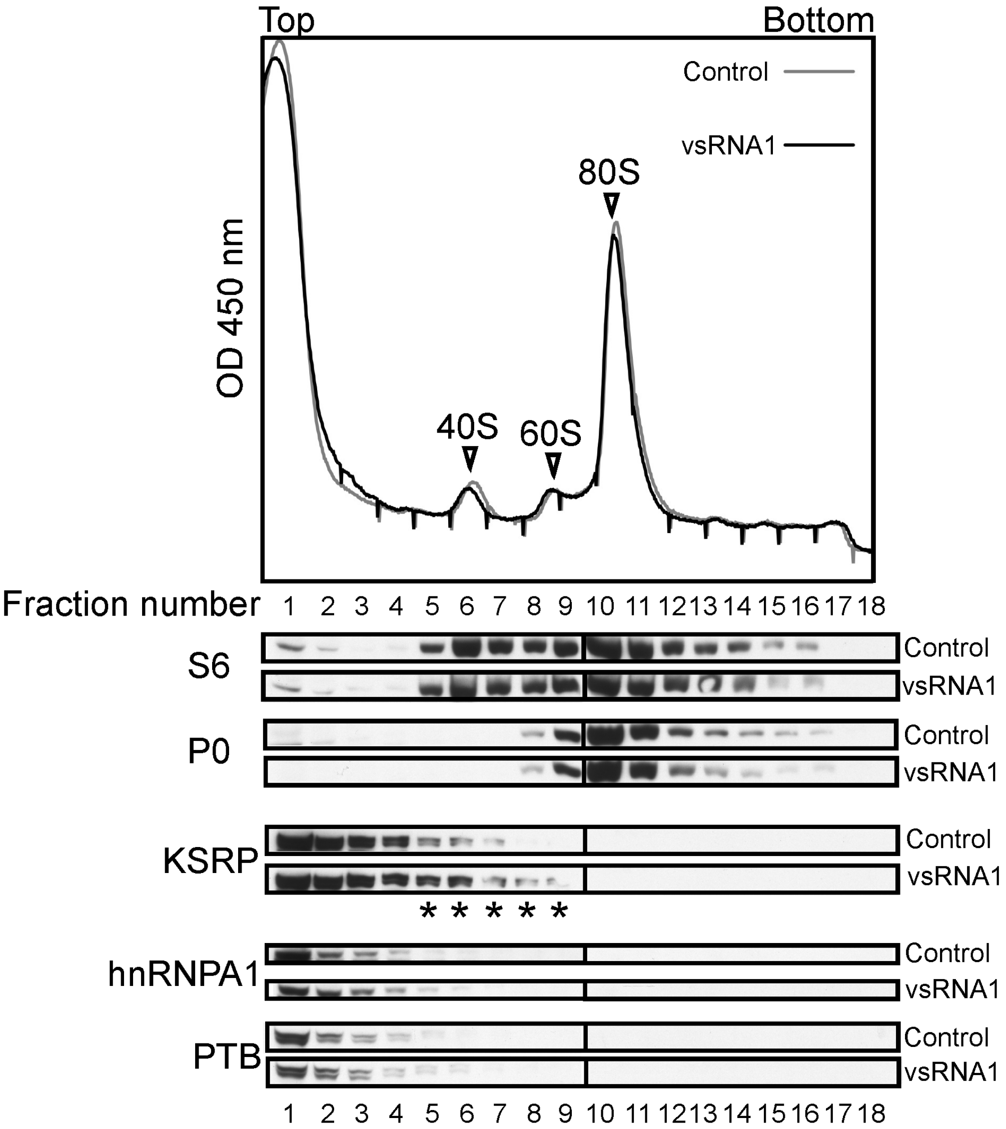

Figure S12. Effects of vsRNA1 on polysomal profiling. RD cells were transfected with scrambled (control) RNA or vsRNA1 mimic, and then harvested at 6 h post-infection. Cell extracts were sedimented on 7%-47% sucrose gradient and were fractionated. Fractions were collected and the absorbance at OD 254 nm was measured. S6 and P0 ribosomal proteins and ITAFs of EV71 IRES, including KSRP, hnRNPA1, PTB were analyzed by western blotting. The stars indicate the differing cosedimentation with ribosomal subunit proteins for KSRP in control RNA and vsRNA1-transfected cells.
